# In Vitro and In Vivo Anti-Phytopathogenic Fungal Activity of a Culture Extract of the Marine-Derived Fungus, *Aspergillus unguis* KUFA 0098, and Its Major Depsidone Constituents

**DOI:** 10.3390/md23120461

**Published:** 2025-11-29

**Authors:** Decha Kumla, Diana I. C. Pinho, Emília Sousa, Tida Dethoup, Luis Gales, Sharad Mistry, Artur M. S. Silva, Anake Kijjoa

**Affiliations:** 1 Faculty of Pharmaceutical Sciences, Burapha University, 169 Long Had Bangsaen Rd., Chonburi 20131, Thailand; decha.ku@go.buu.ac.th; 2 Laboratório de Química Orgânica e Farmacêutica, Departamento de Ciências Químicas, Faculdade de Farmácia, Universidade do Porto and CIIMAR, Rua de Jorge Viterbo Ferreira 228, 4050-313 Porto, Portugal; up201706356@edu.ff.up.pt (D.I.C.P.); esousa@ff.up.pt (E.S.); 3 Department of Plant Pathology, Faculty of Agriculture, Kasetsart University, Bangkok 10240, Thailand; 4 School of Medicine and Biomedical Sciences Abel Salazar (ICBAS) and Instituto de Biologia Molecular e Celular (i3S-IBMC), Rua de Jorge Viterbo Ferreira, 228, 4050-313 Porto, Portugal; lgales@ibmc.up.pt; 5 Department of Chemistry, University of Leicester, University Road, Leicester LE 7 RH, UK; scm11@leicester.ac.uk; 6 Departamento de Química & QOPNA, Universidade de Aveiro, 3810-193 Aveiro, Portugal; artur.silva@ua.pt; 7 School of Medicine and Biomedical Sciences Abel Salazar (ICBAS) and CIIMAR, Rua de Jorge Viterbo Ferreira, 228, 4050-313 Porto, Portugal

**Keywords:** *Aspergillus unguis*, marine-derived fungus, depsidones, phytopathogenic fungi, leaf spot disease, leaf blast disease, greenhouse experiments

## Abstract

The crude ethyl acetate extract of the culture of a marine sponge-associated fungus, *Aspergillus unguis* KUFA 0098, was tested for its capacity to inhibit the growth of ten phytopathogenic fungi, *viz*. *Alternaria brassicicola*, *Bipolaris oryzae*, *Colletotrichum capsici*, *Curvularia oryzae*, *Fusarium semitectum*, *Lasiodiplodia theobromae*, *Phytophthora palmivora*, *Pyricularia oryzae*, *Rhizoctonia oryzae*, and *Sclerotium roflsii.* At a concentration of 1 g/L, the crude extract was most active against *P. palmivora*, causing the highest growth inhibition (55.32%) of this fungus but inactive against *R. oryzae* and *S. roflsii*. At a concentration of 10 g/L, the crude extract completely inhibited the growth of most of the fungi, except for *L. theobromae*, *R. oryzae*, and *S. roflsii*, with 94.50%, 74.12%, and 67.80% of inhibition, respectively. The crude extract of *A. unguis* KUFA 0098 exhibited growth-inhibitory effects against *B. oryzae* and *P. oryzae*, causative agents of brown leaf spot disease and leaf blast disease, respectively, on rice plant var. KDML105, under greenhouse conditions. Chromatographic fractionation and purification of the extract led to the isolation of four previously described depsidones, *viz.* unguinol (**1**), 2-chlorounguinol (**2**), 2,4-dichlorounguinol (**3**), and folipastatin (**4**), as well as one polyphenol, aspergillusphenol A (**5**). The major compounds, i.e., **1**, **2**, and **4**, were tested against the ten phytopathogenic fungi. Compounds **1** and **4** were able to inhibit growth of most of the fungi, except *L. theobromae, R. oryzae*, and *S. roflsii*. Compound **1** showed the same minimum inhibitory concentration (MIC) values as that of carbendazim against *A. brassicicola*, *C. capsici*, *C. oryzae*, and *P. oryzae*, while compound **4** showed the same MIC values as that of carbendazim against only *C. capsici* and *P. oryzae*. Compound **2** was not active against all of the ten phytopathogenic fungi tested.

## 1. Introduction

Synthetic fungicides are still popular and most widely used by farmers to control plant diseases, caused by plant pathogenic fungi, due to their high efficacy and persistence [1]. However, the drawback of synthetic fungicides is that their continuous and excessive use can lead to high resistance of the pathogens and cause negative impacts on people’s health, environment, as well as non-target organisms [2]. Since pathogenic fungi are responsible for approximately 70–80% of total agricultural product loss, which is a great concern for countries with economies heavily reliant on agriculture [3], managing an agricultural system in an eco-friendly and profitable manner by using fungicidal natural products that can minimize fungal infections in an agricultural ecosystem is a challenging task for sustainable agriculture [2]. Thus, the development and manufacture of effective, safe-to-humans, and environmentally friendly fungicides have always been the most desirable goal [4]. 

One of the most popular approaches is the use of botanical fungicides since plant extracts normally contain a variety of compounds that can efficiently inhibit the growth of phytopathogenic fungi, probably through synergistic effects. Furthermore, the different modes of action of natural products in plant extracts can also avoid the emergence of drug resistance [5]. Despite these great advantages, botanical fungicides still have a number of limitations. First, they are less effective than synthetic fungicides and are not always promptly available on the market when needed. Moreover, since they are more easily degradable in the environment, they are less persistent when compared to synthetic fungicides. Other hurdles include variation in the chemical compositions of plants that are collected in different geographical locations and climatic conditions, and the need to obtain a large quantity of biomass of selected plants for large-scale production for commercialization [6]. 

Another interesting nature-based approach is the use of endophytic fungi to protect plants against phytopathogens [7]. This approach has no risk of evolution of pathogen resistance compared to agrochemicals [8]. From this perspective, endophytic fungi can be explored for their use against phytopathogenic fungi since they have a capacity to produce a myriad of structurally diverse and biologically active secondary metabolites to protect their hosts from pathogenic microorganisms [9]. Despite this logic, no compounds produced by fungi have been used as commercial fungicides until now. However, it is important to mention that the syntheses of the strobilurin fungicides were inspired by the natural product, strobilurin A, which was isolated from *Strobilurus tenacellus*, a type of basidiomycete fungus that is often found on decaying wood [10]. These synthetic strobilurin fungicides and strobilurin A share a common structural feature, i.e., the methyl ester of β-methoxyacrylic acid [11]. 

An extensive research program with the aims of preparing analogs of natural products with high levels of activity and suitable physical properties for an agricultural fungicide has led to the development of several strobilurin fungicides, including azoxystrobin, pyraclostrobin, fluoxastrobin, kresoxim-methyl, trifloxystrobin, picoxystrobin, mandestrobin, and metominostrobin [12]. Strobilurin fungicides are also referred to as QoI fungicides since they specifically bind to the cytochrome bc_1_ complex, thereby preventing electron transport and energy production via oxidative phosphorylation [13]. This unique and non-target specific mechanism made these fungicides broad-spectrum, rapid, and highly efficient germicidal activities. Moreover, their cost-effective and rapid degradation during plant metabolism have contributed to the large-scale use of these fungicides [14]. Strobilurin fungicides are globally used to combat white mold, rot, early and late leaf spot, rusts, and rice blast [15]. Among the strobilurin fungicides, azoxystrobin shares its biochemical mode of action with natural strobilurins. Moreover, azoxystrobin is not cross-resistant to other site-specific fungicides, such as benzimidazoles, DMIs, or phenylamides. This fungicide provides systemic, protectant, and curative action and is sold under various trade names, such as Abound, Amistar, and Quadris, which represents an effective new tool for the management of resistance, which is frequently a significant factor in the choice of fungicide products. Despite these advantages, long-term use of strobilurins has raised a serious public health concern due to their environmental contamination and non-target toxicity. Moreover, since strobilurins act on one specific site of fungal pathogens, they are susceptible to resistance. Consistently, several resistance genes from fungi treated with strobilurin have been reported [16,17,18], suggesting that strobilurins can potentially cause long-term adverse effects to the ecosystem.

Intriguingly, due to the rich chemical diversity of the marine environment, much more attention has been paid to the screening of marine-derived fungi than their terrestrial counterparts in recent years [19]. For this reason, in our program of the exploitation of marine natural products for sustainable agriculture, we have screened the growth-inhibitory activity of the ethyl acetate (EtOAc) extracts of cultures of various marine-derived fungi, collected from the Gulf of Thailand, against ten phytopathogenic fungi, which cause diseases for important economic crop plants in Thailand. For example, the crude extract of a marine sponge-associated fungus, *Talaromyces tratensis* KUFA 0091, was tested against *Alternaria padwickii* (Ganguly) M.B. Ellis, *Bipolaris oryzae* (Breda de Haan) Shoem., *Curvularia lunata* (Wakk) Boedjin., and *Fusarium moniliforme* J. Sheld, the causative agents of rice brown spot and dirty panicle, in vitro and in greenhouse conditions. The crude extract completely inhibited the radial growth of all tested pathogens at a concentration of 10 g/L and significantly inhibited the mycelial growth of *B. oryzae* by 53% at 1 g/L. Under greenhouse conditions, the highest reductions in the incidences of brown spot and dirty panicle were 56.74% and 60% when treated with the crude extract at 5 g/L [20]. The crude extract of this fungus, at 5 g/L, was the most effective in controlling brown leaf spot, with disease reduction in the field of 45.04%, when applied twice. However, at 10g/L, it was moderately effective against rice blast, reducing its incidence by 35.11%, when applied twice under field conditions [21]. In another work, crude extracts of ten marine-derived fungi, *viz*. *Emericella stellatus* KUFA 0208, *Eupenicillium parvum* KUFA 0237, *Neosartorya siamensis* KUFA 0514, *N. spinosa* KUFA 0528, *Talaromyces flavus* KUFA 0119, *T. macrosporus* KUFA 0135, *T. trachyspermus* KUFA 0304, *Trichoderma asperellum* KUFA 0559, *T. asperellum* KUFA 0559, and *T. harzianum* KUFA 0631 were evaluated for their in vitro fungicidal activity against five rice pathogens. However, only the extracts of *E. stellatus* KUFA 0208 and *N. siamensis* KUFA 0514 exhibited the best antifungal activity, causing complete inhibition of the mycelial growth of *A. padwickii*, *Bipalaris oryzae*, *F. semitectum*, *P. oryzae*, and *Rhizoctonia solani* at 10 g/L [22].

In this study, the crude extract of *Aspergillus unguis* KUFA 0098 did not only exhibit interesting growth-inhibitory activity against ten phytopathogenic fungal strains but also showed its preventive and curative effects against leaf brown spot disease, caused by *B. oryzae*, and leaf blast disease, caused by *P. oryzae*, on rice plant var. KMDL105, under greenhouse conditions. Therefore, the chemical constituents of *A. unguis* KUFA 0098 were isolated, characterized, and assayed for the in vitro growth-inhibitory activity against these phytopathogenic fungi.

## 2. Results and Discussion

*Aspergillus unguis* (Aspergillaceae) is a cosmopolitan fungus since it can be isolated from soils [23], plants [24], lichens [25], and marine organisms, including algae [26] and sponges [27]. A review article by Domingos et al. [28], covering the period of 1970-2022, reported the isolation of ninety-seven secondary metabolites from the cultures of *A. unguis*, and a myriad of their biological activities, including antibacterial, antimalarial, larvicidal, and anti-inflammatory activities, enzyme inhibitory activities, and cytotoxicity against some cancer cell lines. Among the secondary metabolites isolated from *A. unguis*, depsidones constituted 30% of the total. Depsidones are an important group of polyketide secondary metabolites since they possess not only diversified structural features but also relevant biological activities [29]. 

Since more attention has been paid to marine-derived fungi as a source of naturally occurring fungicides against phytopathogenic fungi [30,31,32,33], coupled with our interest in searching for bioactive secondary metabolites from marine-derived fungi to combat diseases of the economic crops caused by phytopathogenic fungi [34], we have tested the effect of the crude EtOAc extract of the culture of *A. unguis* KUFA 0098, isolated from a marine sponge *Hyrtios erectus*, against phytopathogenic fungi.

The crude EtOAc extract of the culture of *A. unguis* KUFA 0098 was assayed for its capacity to inhibit the growth of ten plant pathogenic fungi, which are causative agents of economic crops in Thailand, *viz*. *Alternaria brassicicola* KUFA 1031 (black spot of Chinese Kale), *B. oryzae* KUFA 1032 (brown spot of rice), *Colletotrichum capsici* KUFA 1033 (anthracnose of chili), *Curvularia oryzae* KUFA 1034 (leaf spot of rice), *F. semitectum* KUFA 1035 (dirty panicle of rice), *Lasiodiplodia theobromae* KUFA 1036 (fruit rot of durian), *Phytophthora palmivora* KUFA 1037 (root and stem rot of durian), *P. oryzae* KUFA 1038 (rice blast), *Rhizoctonia oryzae* KUFA 1039 (sheath blight of rice), and *Sclerotium roflsii* KUFA 1040 (stem rot of bean). At a concentration of 1g/L, the crude EtOAc extract did not inhibit the mycelial growth of *R. oryzae* and *S. roflsii* but inhibited the mycelial growth of the rest of the tested phytopathogenic fungi to less than 50%, except for *P. palmivora*, for which the inhibition was 55.32%. At a concentration of 10 g/L, the crude EtOAc extract completely inhibited the mycelial growth of seven phytopathogenic fungi, *viz. A. brassicicola*, *B. oryzae*, *C. capsici*, *C. oryzae*, *F. semitectum*, *P. palmivora*, and *P. oryzae*; however, it was less active toward *R. oryzae* and *S. roflsii*, showing inhibition of 74.12 and 67.80% of their mycelial growth, respectively (Table 1). 

Since the crude EtOAc extract of *A. unguis* KUFA 0098 showed 100% inhibition of the mycelial growth of *B. oryzae* and *P. oryzae* at a concentration of 10g/L, and 27.46 and 20. 67% at a concentration of 1 g/L, the crude extract was evaluated for its capacity to control brown leaf spot disease (caused by *B. oryzae*) and the leaf blast disease (caused by *P. oryzae*) on rice plants var. KMDL105 under greenhouse conditions. The reasons why we selected these two rice diseases are not only because rice is the most valuable commodity in the world but also because it takes a reasonable amount of time to perform experiments with rice plants in greenhouse conditions.

**Figure 1:**
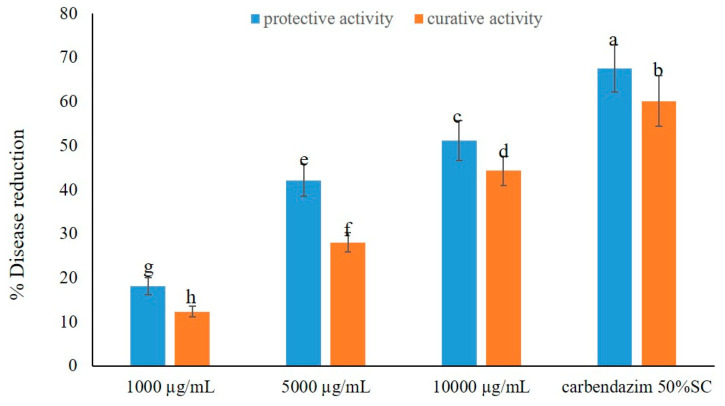
Effects of the crude extract of *A. unguis* KUFA 0098 against brown leaf spot disease under greenhouse conditions. Means ± SD, followed by the same letter, do not significantly differ at *p* < 0.05 when analyzed using Duncan’s test of one-way ANOVA.

**Figure 2:**
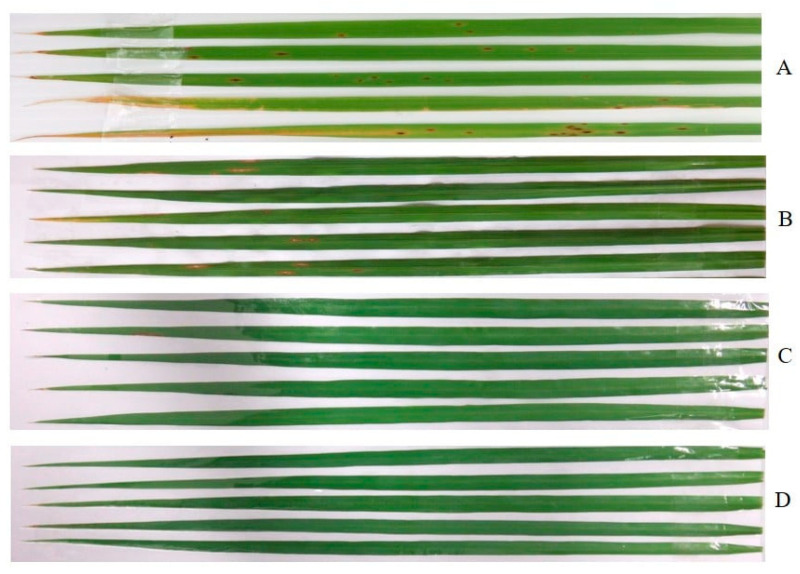
Effects of the crude extract of *A. unguis* KUFA 0098 against brown leaf spot disease under greenhouse conditions when applied before pathogen inoculation. A = control; B = 1000 µg/mL; C = 5000 µg/mL; and D = 10,000 µg/mL.

The crude EtOAc extract showed its capacity to control brown leaf spot disease in a dose-dependent manner, and without phytotoxicity (Figure 1). Moreover, the crude extract exhibited higher protective activity than curative activity in disease reduction. Application of the crude extract at the highest dose, i.e., at 10,000 µg/L, displayed remarkable fungicidal activity, causing 51.23 and 44.32% reduction in protective and curative activity tests, respectively. Moreover, even at a concentration of 5000 µg/L, the crude extract showed moderate activity against the disease, reducing the disease incidence by 42.16 and 28.09%, respectively. However, at a concentration of 1000 µg/L, the crude extract displayed low activity, causing less than 20% of disease reduction in both activity tests. Tellingly, the application of a standard fungicide, carbendazim, exerted a higher disease reduction in both activity tests, *viz*. *ca*. 70% in protective activity and *ca.* 60% in curative activity (Figure 1). The effects of the crude extract of *A. unguis* KUFA 0098 against brown leaf spot disease under greenhouse conditions are shown in Figure 2.

**Figure 3:**
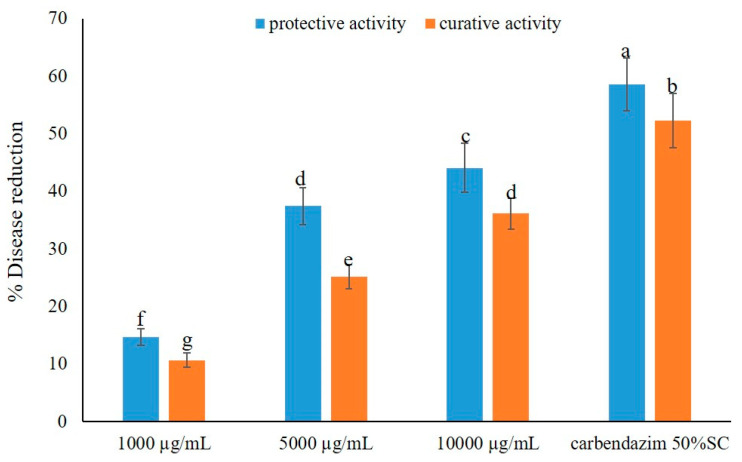
Effects of the crude extract of *A. unguis* KUFA 0098 against leaf blast disease under greenhouse conditions. Means ± SD, followed by the same letter, do not significantly differ at *p* < 0.05 when analyzed using Duncan’s test of one-way ANOVA.

**Figure 4:**
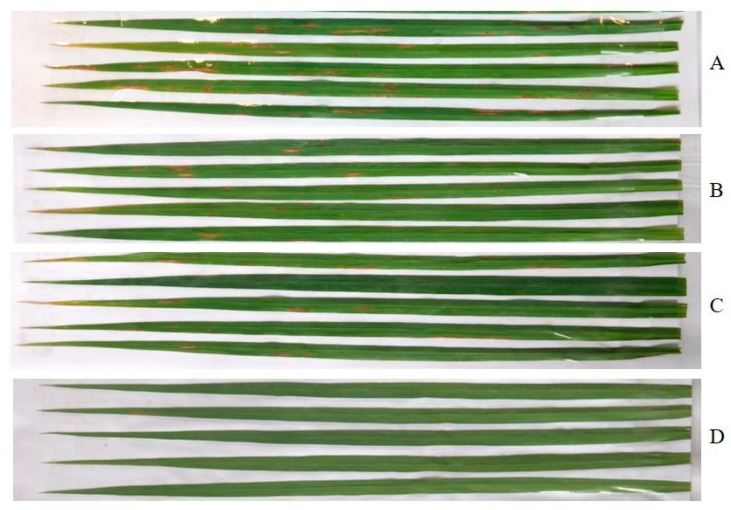
Effects of the crude extract of *A. unguis* KUFA 0098 against leaf blast disease under greenhouse conditions when applied before pathogen inoculation. A = control; B = 1000 µg/mL; C = 5000 µg/mL; and D =10,000 µg/mL.

The effects of the crude extract against the leaf blast disease, under greenhouse conditions, were similar to those of brown leaf spot disease. The crude extract showed a reduction in leaf blast disease in a dose-dependent manner and displayed more potent protective activity than curative activity. However, it exhibited lower activity against the leaf blast test when compared to the brown leaf spot test. Application of the crude extract at the highest dose, i.e., 10,000 µg/mL, exhibited potent activity, causing 44.09 and 36.17% disease reduction in the protective and curative activity tests, respectively. Application of the crude extract at lower concentration, at 5000 µg/mL, showed moderate activity against the disease, reducing the disease incidence by 37.43% and 25.15%, respectively. However, the extract displayed low activity when applied at a concentration of 1000 µg/mL, causing less than 15% of disease reduction in both activity tests. Similarly to brown leaf spot, the application of a fungicide, carbendazim, also exerted a higher disease reduction in both activity tests, *viz. ca.* 60% in protective activity and *ca.* 55% in curative activity (Figure 3). The effects of the crude extract of *A. unguis* KUFA 0098 against leaf blast disease under greenhouse conditions are shown in Figure 4.

**Figure 5:**
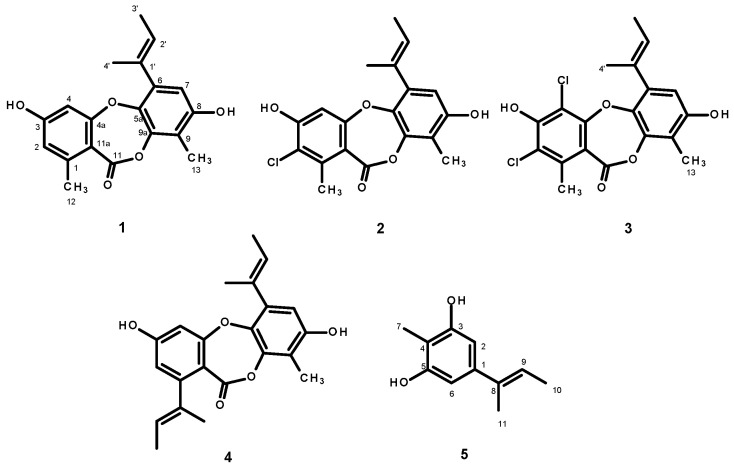
Structures of unguinol (**1**), 2-chlorounguinol (**2**), 2,4-dichlorounguinol (**3**), folipastatin (**4**), and aspergillusphenol A (**5**), isolated from the culture extract of *Aspergilus unguinol* KUFA 0098.

Since the crude EtOAc extract showed promising results, inhibiting completely the growth of seven out of ten phytopathogenic fungi, at a concentration of 10g/L (Table 1), we proceeded with the isolation and characterization of the constituents of the crude EtOAc extract to isolate the compounds and to test them individually against these ten phytopathogenic fungi in order to identify the compounds responsible for the activity of the crude extract. The EtOAc crude extract was first partitioned to CHCl_3_, and after evaporation of the solvent by reduced pressure, the crude CHCl_3_ extract was obtained. Fractionation of the CHCl_3_ portion by column chromatography of silica gel occurred, followed by purification by crystallization, preparative TLC and Sephadex LH-20 column, furnished unguinol (**1**) [35], 2-chlorounguinol (**2**) [36], and folipastatin (**4**) [37] as major compounds (with yields of 1.72, 1.43, and 1.21%, respectively), together with 2,4-dichlorounguinol (**3**, 0.05%) [37], and aspergillusphenol A (**5**, 0.02%) [37] as minor compounds (Figure 5). 

The structures of **1**-**5** were elucidated by an interpretation of their 1D (^1^H, ^13^C, DEPT) and 2D NMR (COSY, HSQC and HMBC) (Figures S1–S25 and Tables S1–S5), as well as a comparison of their NMR data with those previously reported. The structures of **2** and **3** were confirmed by (+)-HRESIMS spectra (Figures S26 and S27). The crystal structures of **1**, **2**, **4**, and **5** were obtained, for the first time, by an X-ray diffractometer equipped with CuKα radiation, and their Ortep views are shown in Figure 6. 

Since the three depsidones, *viz*. **1**, **2**, and **4**, were isolated in high quantities from the crude EtOAc extract of *A. unguis* KUFA 0098, they were assayed for in vitro antifungal activity against the ten phytopathogenic fungi, using carbendazim as a positive control. Table 2 shows that **1** was the most active compound, showing similar growth-inhibitory activity to that of the positive control, carbendazim (MIC = 125 μg/mL), against *A. brassicicola, C. capsici, C. oryzae*, and *P. oryzae*, (MIC = 125 μg/mL), and it was two folds less active than carbenazim against *B. oryzae* (MIC = 125 μg/mL), *F. semitectum* (MIC = 250 μg/mL), and *P. palmivora* (MIC = 125 μg/mL) but inactive (MIC ˃ 500 μg/mL) against *L. theobromae, R. oryzae*, and *S. roflsii*. On the other hand, **4** was more active towards less phytopathogenic fungi than **1**. Compound **4** showed comparable inhibitory activity against *C. capsici* and *P. oryzae* (MIC = 125 μg/mL) with that of carbendazim (MIC = 125 μg/mL), but it was two folds less active than carbendazim against *P. palmivora* (MIC = 125 μg/mL) and *F. semitectum* (MIC = 250 μg/mL), and inactive against the rest of the fungi (MIC = 500 ˃ μg/mL). Intriguingly, **2** was inactive against all the phytopathogenic fungi tested (MIC = 500 ˃ μg/mL) (Table 2).

Interestingly, the introduction of bulky groups to the depsidone scaffold slightly affected the growth-inhibitory activity of the depsidones since **4**, an analog of **1**, with the but-2-en-2-yl group on C-1 and the methyl group on C-4, displayed a similar growth-inhibitory activity to that of **1**, except for *A. brassicicola* and *C. oryzae.* On the other hand, the introduction of a chlorine atom to C-2 of **1** dramatically decreased its growth-inhibitory activity, as evidenced by the lack of activity of **2** against all the phytopathogenic fungi tested. It is interesting to note that all of the three depsidones did not inhibit the growth of three phytopathogenic fungi, *viz. L. theobromae*, *R. oryzae*, and *S. roflsii.*

Intriguingly, our findings are in contrast to those reported by Sadorn et al., who found that the chlorodepsidones, *viz.* emerguisin A (7-chloro), 2-chlorounguinol (2-chloro), and nornidulin (2,4,7-trichloro), isolated from the culture of the endophytic fungus, *A. unguis* BCC54176, which was obtained from a leaf of coriander, showed anti-phytopathogenic activity against *C. acutatum*, with the same MIC value (3.13 μg/L), which is two folds less active than the positive control, amphotericin B (MIC = 1.56 μg/L). On the other hand, nornidulin (MIC = 25 μg/L) and 2-chlorounguinol (MIC = 12.5 μg/L) were less active against *A. brassicicola*, while emerguisin A was inactive (MIC ˃ 50 μg/L) against this pathogenic fungus. Conversely, folastatin was inactive against both *A. brassicicola* and *C. acutatum*, while unguinol was inactive against *C. acutatum* but slightly active against *A. brassicicola* [24].

The discrepancies of the results obtained by us in this work and by Sadorn et al. [24] could be due to either the different methods used to determine the growth-inhibitory activity of the compounds or the source and type of the phytopathogenic fungal strains. While the CLSI method is used in this work to determine MICs of the compounds, with carbendazim as a positive control, Sadorn et al. [24] used the 5(6)-carboxyfluorescein diacetate (CFDA) fluorometric assay, with amphotericin B as a positive control. The source of the phytopathogenic fungi can also influence the obtained results. While Sadorn et al. [24] used commercially available strains, i.e., *A. brassicicola* (BCC42724) and *C. acutatum* (BCC58146), we used the strains of the pathogenic fungi isolated from the diseased host plants with their pathogenicity confirmed in our laboratory.

## 3. Experimental Section

### 3.1. General Experimental Procedures

^1^H and ^13^C NMR spectra were recorded at ambient temperature on a Bruker AMC instrument (Bruker Biosciences Corporation, Billerica, MA, USA) operating at 300 and 75 MHz, respectively. High-resolution mass spectra (HRMS) were measured with a Waters Xevo QToF mass spectrometer (Waters Corporations, Milford, MA, USA) coupled to a Waters Aquity UPLC system. A Merck (Darmstadt, Germany) Silica gel GF_254_ was used for preparative TLC, and a Merck Si gel 60 (0.2–0.5 mm) was used for column chromatography. LiChroprep silica gel and Sephadex LH-20 were used for column chromatography.

### 3.2. Fungal Strain

The strain KUFA 0098 was isolated from the marine sponge, *Hyrtios erectus*, which was collected by scuba diving at a depth of 15-20 m, from a coral reef at Samae San Island (12°34′36.64″ N 100°56′59.69″ E), Chonburi province, Thailand, in April 2016. The sponge was washed with sterilized seawater three times, dried on a sterile filter paper under sterile aseptic condition, and cut into small pieces (5 × 5 mm). Four pieces of the sponges were placed on Petri dish plates containing 15 mL of potato dextrose agar (PDA) medium mixed with 300 mg/L of streptomycin sulfate and incubated at room temperature for 7 d. The hyphal tips emerging from the pieces of sponges were individually transferred onto a PDA slant and maintained as pure cultures at Kasetsart University Fungal Collection, Department of Plant Pathology, Faculty of Agriculture, Kasetsart University, Bangkok, Thailand.

The fungal strain KUFA 0098 was identified as *Aspergillus unguis*, based on morphological characteristics. This identification was confirmed by molecular techniques using ITS primers. DNA was extracted from young mycelia following a modified Murray and Thompson method [38]. The universal primer pairs ITS1 and ITS4 were used for ITS gene amplification [39]. PCR reactions were conducted on Thermal Cycler, and the amplification process consisted of initial denaturation at 95 °C for 5 min, 34 cycles at 95 °C for 1 min (denaturation), at 55 °C for 1 min (annealing), and at 72 °C for 1.5 min (extension), followed by final extension at 72 °C for 10 min. PCR products were examined by agarose gel electrophoresis (1% agarose with 1×TBE buffer) and visualized under UV light after staining. DNA sequencing analyses were performed using the dideoxyribonucleotide chain termination method [40] by Macrogen Inc. (Seoul, Republic of Korea). The DNA sequences were edited using FinchTV software (version 1.4) and submitted to the BLAST program for alignment and compared with that of fungal species in the NCBI database (http://www.ncbi.nlm.nih.gov/ accessed on 20 December 2020). Its gene sequences were deposited in GenBank with the accession number PV719693. 

### 3.3. Extraction and Isolation of the Compounds

The mycelial plugs of *A. unguis* KUFA 0098 were transferred into 500 mL Erlenmeyer flasks containing 200 mL of potato dextrose broth (PDB) and incubated at room temperature on a rotary shaker at 120 rpm for 7 d to prepare mycelial suspension. Fifty 1 L Erlenmeyer flasks, each containing 300 g of sterile cooked rice, were then inoculated with 20 mL of mycelial suspension in each flask and incubated at room temperature for 30 d, after which 500 mL of EtOAc was added to each flask and macerated for 7 d and then filtered with filter paper (Whatman No.1) to produce the organic solutions, which were combined and evaporated under reduced pressure to furnish a crude EtOAc extract (300 g). The crude EtOAc extract was then dissolved in EtOAc (300 mL) and washed with H_2_O (3 × 500 mL), and the EtOAc solution was dried over anhydrous Na_2_SO_4_, filtered, and evaporated under reduced pressure to obtain 270.2 g of the crude EtOAc extract. The crude EtOAc extract was then dissolved in 500 mL of CHCl_3_ and filtered. The CHCl_3_ solution was evaporated under reduced pressure to obtain 157.3 g of a crude CHCl_3_ extract. The crude CHCl_3_ extract was applied on a column of silica gel (400 g) and eluted with mixtures of petrol/CHCl_3_ and CHCl_3_/Me_2_CO, wherein 250 mL fractions (Frs) were collected as follows: Frs 1–83 (CHCl_3_–petrol, 7:3), 84–184 (CHCl_3_–petrol, 9:1), 185–186 (CHCl_3_), 187–232 (CHCl_3_-Me_2_CO, 9:1), and 233–255 (CHCl_3_-Me_2_CO, 8:2). Frs 87–115 were combined (4.42 g) and crystalized in CHCl_3_ to give 1.21 g of **2**. Frs.116–146 were combined (4.45 g) and crystalized in CHCl_3_ to give 1.18 g of white crystals of **2**. Frs. 147–184 were combined (2.31 g) and crystalized in CHCl_3_ to give 912.2 mg of **2**. Frs. 185–191 were combined (1.12 g) and crystalized in CHCl_3_ to give 543.8 mg of **2**. The mother liquor of Frs 87–115 (3.35 g) was applied on a column chromatography of silica gel (30 g) and eluted with mixtures of petrol and CHCl_3_, wherein 150 mL subfractions (sfrs) were collected as follows: sfrs 19–41 (CHCl_3_–petrol, 3:7), sfrs 42–46 (CHCl_3_–petrol, 3:7), sfrs 47–53 (CHCl_3_–petrol, 3:7), sfrs 54–61 (CHCl_3_–petrol, 3:7), sfrs 62–79 (CHCl_3_–petrol, 5:5), sfrs 80–87 (CHCl_3_–petrol, 7:3), and sfrs 88–99 (CHCl_3_–petrol, 9:1). Sfrs 62–87 were combined (383.6 mg) and purified by preparative TLC of silica gel G_254_ and eluted with CHCl_3_-Me_2_CO-petrol, 90:5:5, to produce 12.2 mg of **3**. The mother liquor of frs.116 was applied on a column chromatography of silica gel (30 g) and eluted with mixtures of petrol and CHCl_3_, wherein 150 mL sfrs were collected as follows: sfrs 5–14 (CHCl_3_–petrol, 3:7), sfrs 15–68 (CHCl_3_–petrol, 3:7), sfrs 69–79 (CHCl_3_–petrol, 5:5), and Sfrs 80–92 (CHCl_3_–petrol, 7:3). Sfrs 5–41 were combined and crystallized in CHCl_3_ to give 483.3 mg of **5**. Frs. 192 and 193 were combined (9.54 g) and crystalized in CHCl_3_ to give 3.28 g of **4**. Frs 194–198 were combined (7.25 g) and crystallized in CHCl_3_ to give 4.64 g of **1**.

### 3.4. X-Ray Crystallography 

Single crystals were mounted on cryoloops using paratone, X-ray diffraction data were collected at 293 K with a Gemini PX Ultra equipped with CuK_α_ radiation (λ = 1.54184 Å). The structures were solved by direct methods using SHELXS-97 and refined with SHELXL-97 [41]. Non-hydrogen atoms were refined anisotropically. Hydrogen atoms were either directly found from difference Fourier maps and refined freely with isotropic displacement parameters or were placed at their idealized positions using appropriate *HFIX* instructions in SHELXL and included in subsequent refinement cycles. Full details of the data collection and refinement and tables of atomic coordinates, bond lengths and angles, and torsion angles are deposited with the Cambridge Crystallographic Data Centre.

#### 3.4.1. Unguinol (**1**)

The crystal was triclinic, space group P-1, with a unit cell volume of 1898.8(2) Å^3^. The unit cell dimensions were *a* = 11.7197(6) Å, *b* = 12.0288(9) Å, and *c* = 15.3894(6) Å, with angles α = 82.936(4)°, β = 87.858(4)°, and γ = 61.903(6)° (uncertainties in parentheses). The asymmetric unit contained one molecule of the compound and one solvent molecule of chloroform. The refinement converged with R (all data) = 14.06% and wR2 (all data) = 36.74%. Cambridge Crystallographic Data Centre deposition number CCDC 2452372.

#### 3.4.2. 2-Chlorounguinol (**2**)

The crystal was monoclinic, space group P 2_1_/*c*, with a unit cell volume of 1844.63(17) Å^3^. The unit cell dimensions were *a* = 9.9458(5) Å, *b* = 18.7156(7) Å, and *c* = 11.0150(5) Å, with β = 115.886(6)° (uncertainties in parentheses). The asymmetric unit contained one molecule of the compound and one water molecule. The refinement converged with R (all data) = 9.96% and wR2 (all data) = 26.12%. Cambridge Crystallographic Data Centre deposition number CCDC 2452435.

#### 3.4.3. Folipastatin (**4**)

The crystal was triclinic, space group P-1, with a unit cell volume of 2503.9(2) Å^3^. The unit cell dimensions were *a* = 9.4348(5) Å, *b* = 14.8071(6) Å, and *c* = 19.8438(8) Å, with angles α = 71.171(4)°, β = 83.665(4)°, and γ = 72.630(4)° (uncertainties in parentheses). The asymmetric unit contained two molecules of the compound. The refinement converged with R (all data) = 17.16% and wR2 (all data) = 40.88%. Cambridge Crystallographic Data Centre deposition number CCDC 2451960.

#### 3.4.4. Aspergillusphenol A (**5**)

The crystal was monoclinic, space group P 2_1_/*n*, with a unit cell volume of 973.31(7) Å^3^. The unit cell dimensions were *a* = 8.0419(4) Å, *b* = 12.7973(4) Å, and *c* = 9.9934(4) Å, with β = 108.850(5)° (uncertainties in parentheses). The asymmetric unit contained one molecule of the compound. The refinement converged with R (all data) = 6.72% and wR2 (all data) = 14.84%. Cambridge Crystallographic Data Centre deposition number CCDC 2451810.

### 3.5. In Vitro Antifungal Activity Bioassays Against Plant Pathogenic Fungi

#### 3.5.1. Pathogen Isolation and Pathogenicity Test

All phytopathogen strains, *viz*. *Alternaria brassicicola* KUFA 1031, *Bipolaris oryzae* KUFA 1032, *Colletotrichum capsici* KUFA 1033, *Curvularia oryzae* KUFA 1034, *Fusarium semitectum* KUFA 1035, *Lasiodiplodia theobromae* KUFA 1036, *Phytophthora palmivora* KUFA 1037, *Pyricularia oryzae* KUFA 1038, *Rhizoctonia oryzae* KUFA 1039, and *Sclerotium roflsii* KUFA1040, were isolated from diseased host plants and identified by morphological analysis. Their pathogenicity test was conducted according to the method described by Agrios [42] on the host plants under greenhouse conditions, as previously reported [20,43,44].

#### 3.5.2. In Vitro Antifungal Activity of the Crude EtOAc Extract of *A. unguinol* KUFA 0098 Against Plant Pathogenic Fungi

The in vitro growth-inhibitory activity of the crude EtOAc extract of *A. unguis* KUFA 0098, against ten plant pathogenic fungi, was evaluated using the dilution plate technique, according to the previously described method [20]. Briefly, the crude EtOAc extract (1.0 g) was dissolved in 1 mL of dimethyl sulfoxide (DMSO) and serially diluted with 9 mL of H_2_O to obtain stock solutions of 100 and 10 g/L. In total, 1 mL of each stock solution was added to 9 mL of warm PDA, thoroughly mixed by a vortex mixer, and then poured into Petri dishes to give medium plates amended with the crude extract with concentrations of 10g/L and 1 g/L, respectively. Each pathogen strain was cultured on a PDA for 7 d, at 28 ± 2 °C, and a mycelial plug of 0.5 cm in diameter of each pathogen was placed on the center of the PDA plates containing the crude extract, which were incubated at room temperature for 14 d. The PDA plate void of the crude extract was used as a negative control. The inhibition levels were calculated using the formula [(x − y)/x] × 100, where x is a diameter of the colony growth of the plant pathogenic fungi in the negative control, and y is a diameter of the colony growth of the plant pathogenic fungi in the presence of the crude extract. Each treatment was performed with five replications and repeated three times independently. The mean inhibition levels and standard deviations were calculated from five replications and three repetitions. Data were subjected to analysis of variance, and means were subsequently compared using Duncan’s multiple range test (*p* ˂ 0.05) in the SPSS version 19 statistical program (IBM Corporation, Somers, NY, USA).

#### 3.5.3. In Vitro Antifungal Assays of Unguinol (**1**), 2-Chlorounguinol (**2**), and Folipastatin (**4**) Against Plant Pathogenic Fungi

The antifungal activity of unguinol (**1**), 2-chlorounguinol (**2**), and folipastatin (**4**), major secondary metabolites isolated from the culture extract of the marine-derived *A. unguis* KUFA 0098, against the plant pathogens was assayed according to the CLSI recommendation by testing the minimum inhibitory concentrations (MICs) [45]. Each pathogen strain was cultured on PDA for 14 d at 28 ± 2 °C. The spores of the pathogenic fungi were collected by gently scraping from the mycelium by a sterile loop to add to the PDB and adjust it to 10^6^ spores/mL with PDB. In total, 1 mg of each compound was dissolved in 100 µL of 10% DMSO, and then 900 µL of distilled H_2_O was added to prepare a stock solution of 1000 µg/L. Two-fold serial dilutions of the stock solution by PDB mixed with a spore suspension provided the tested concentrations at 500, 250, and 125 µg/L. Each treatment was loaded with 200 µL of the compounds per well into 96-well U-shaped sterile polystyrene plates with five wells per treatment and subsequently incubated for 7 d at 25 °C. The lowest concentration that inhibited visible growth was determined as an MIC of the compound. Carbendazim (Sigma-Aldrich®, St. Louis, MI, USA), at the same concentration, was used as a positive control.

### 3.6. Effects of the Crude Extract of A. unguis KUFA 0098 Against Rice Leaf Diseases Under Greenhouse Conditions

#### 3.6.1. Preparation of the Solutions of the Crude Extract of *A. unguis* KUFA 0098

The crude EtOAc extract of *A. unguis* KUFA 0098 (10 g) was thoroughly dissolved in 10 mL of DMSO (Sigma-Aldrich, St. Louis, MO, USA), and then 990 mL of H_2_O, with 1% Tween 80 (Sigma-Aldrich, St. Louis, MO, USA), was added to obtain a concentration of 10,000 µg/L. This solution was diluted with distilled H_2_O to obtain concentrations of 5000 and 1000 µg/L for the experiments.

#### 3.6.2. Preparation of Spore Suspension of *B. oryzae* and *P. oryzae*

*B. oryzae* and *P. oryzae* were cultured separately on PDA plates for 14 d at 28 ± 2 °C. Then, 20 mL of sterile H_2_O was poured into each PDA plate, and spores were gently scraped from mycelium using a sterile glass rod. The spore suspension of each fungus was then passed through three layers of sterile cheesecloth to remove fungal mycelium. Then, the spore suspension was diluted with sterile H_2_O and adjusted using a hematocytometer to obtain 10^6^ spores/mL.

#### 3.6.3. Preparation of Rice Seedlings

Four seven-day-old rice seedlings (rice plant var. KDML 105) of the same height and vigor were transplanted to a plastic pot (25 cm diam. × 22 cm in height) containing sterile clay soil (1500 g). The pots were placed in a greenhouse at 30 ± 2 °C in a completely randomized design (Figure S28). The pots were watered daily. 

#### 3.6.4. Evaluation of the Effects of the Crude Extract of *A. unguis* KUFA 0098 Against Leaf Brown Spot Disease and Leaf Blast Disease Under Greenhouse Conditions 

The preventive and curative activities of the crude extract of *A. unguis* KUFA 0098 against leaf brown spot disease, on rice plant var. KMDL105, were evaluated under greenhouse conditions, following the previously described procedure [20]. 

For the evaluation of the preventative activity, 40-day-old rice plants were sprayed with crude extract solutions at 1000 µg/L, 5000 µg/L, and 10,000 µg/L, 30 mL per pot. Distilled H_2_O and aqueous solution of carbendazim 50% W/V suspension concentrate (SC), at 1.5 mL/L, both containing 1% Tween 80, were used as negative and positive controls, respectively. The sprayed rice plants were left to dry for 2h, after which they were inoculated with 30 mL of spore suspension (containing 1%Tween 80) of *B. oryzae* (in the case of leaf brown spot disease) or *P. oryzae* (in the case of leaf blast disease), at 10^6^ spores/mL/pot. Each treatment consisted of five pots (two replications) with two repetitions. 

For the evaluation of the curative activity, the 30-day-old rice plants were first inoculated with 30 mL of spore suspension (containing 1% Tween 80) of *B. oryzae* at 10^6^ spores/mL/pot. Then, each pot was covered with a transparent plastic bag for 24 h, after which the rice plants were sprayed with the crude extract solutions at 1000 µg/L, 5000 µg/L, and 10,000 µg/L, 30 mL per pot. Distilled H_2_O and aqueous solution of carbendazim 50% W/V SC, at 1.5 mL/L, both containing 1% Tween 80, served as negative and positive controls, respectively. Each treatment consisted of five pots (two replications) with two repetitions. 

Ten days after the inoculation, ten leaves of the rice plants from the middle of the pot were randomly collected for the evaluation of the disease incidence. The numbers of brown leaf spots on each leaf were counted and the average in each treatment was calculated and compared with the control groups. The percentage of disease reduction was calculated as follows:

% disease reduction = [(R^1^ × 100)/R^2^] − 100

where R^1^ is the average of the number of diseased spots per leaf in the plants with treatment, and R^2^ is the average of the number of diseased spots per leaf in the negative control.

### 3.7. Statistical Analysis

All experiments in this study were conducted at least twice. Since there were no significant differences between the repetitions of each experiment, the data from each experiment were pooled and analyzed using the analysis of variance (ANOVA) method. Means were compared using Duncan’s post hoc test (*p* < 0.05) using SPSS version 19 (IBM Corporation, Somers, NY, USA).

## 4. Conclusions

The present work reveals the capacity of the marine-derived fungus, *A. unguis* KUFA 0098, to produce compounds with growth-inhibitory activity against a variety of phytopathogenic fungi that cause diseases in economically important crops both in vitro and in vivo (in greenhouse conditions). Among the three major compounds isolated, unguinol and folipastatin displayed growth inhibition of 80% of the phytopathogenic fungi tested. These results led to the conclusion that these two compounds are responsible for the inhibitory activity of the crude EtOAc extract against eight out of ten phytopathogenic fungi tested and also revealed that depsidone is an important scaffold for anti-phytopathogenic fungal activity. Marine-derived fungi have been proven to be a prolific source of bioactive natural products, and thus the compounds produced by marine-derived fungi can have a great potential to combat not only human diseases but also plant diseases. Therefore, testing compounds produced by marine-derived fungi to search for natural fungicides to control plant diseases is a challenging task. Marine-derived fungal compounds have several advantages over plant-derived compounds. For example, they can be produced on a large scale, using a reactor, and in a shorter amount of time compared to phytochemicals. Moreover, different compounds with the same scaffolds can be obtained by using the OSMAC (One Strain Many Compounds) approach or semi-synthesis, which can lead to obtaining compounds with improved efficacy and less toxicity for use in eco-friendly and sustainable agriculture. 

## Figures and Tables

**Figure 6:**
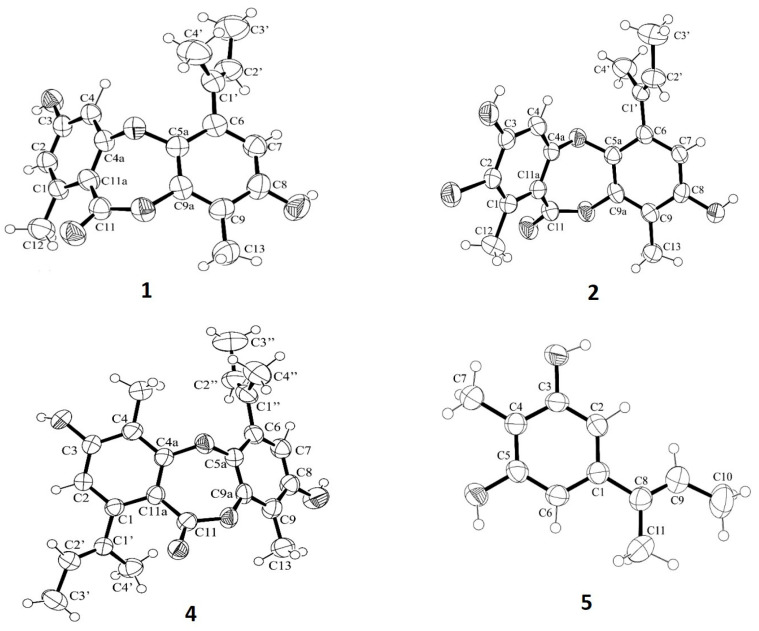
ORTEP views of unguinol (**1**), 2-chlorounguinol (**2**), folipastatin (**4**), and aspergillusphnol A (**5**).

**Table 1:** Effects of the crude EtOAc extract of *Aspergillus unguis* KUFA 0098 on the growth of plant pathogenic fungi.

Plant Pathogen	% Mycelial Growth Inhibition at Concentrations	
	10 g/L	1 g/L
*Alternaria brassicicola* (black spot of Chinese Kale)	100 ± 0 ^a^	25.31 ± 1.24 ^f^
*Bipolaris oryzae* (leaf brown spot of rice)	100 ± 0 ^a^	27.46 ± 0.87 ^e^
*Colletotrichum capsici* (anthracnose of chili)	100 ± 0 ^a^	30.05 ± 1.31 ^d^
*Curvularia oryzae* (leaf spot of rice)	100 ± 0 ^a^	15.64 ± 0.54 ^h^
*Fusarium semitectum* (dirty panicle of rice)	100 ± 0 ^a^	43.71 ± 2.05 ^b^
*Lasiodiplodia theobromae* (fruit rot of durian)	94.50 ± 5.58 ^b^	35.38 ± 2.16 ^c^
*Phytophthora palmivora* (root and stem rot of durian)	100 ± 0 ^a^	55.32 ± 4.25 ^a^
*Pyricularia oryzae* (rice blast)	100 ± 0 ^a^	20.67 ± 0.89 ^g^
*Rhizoctonia oryzae* (sheath blight of rice)	74.12 ± 4.31 ^c^	0 ± 0 ^i^
*Sclerotium roflsii* (stem rot of bean)	67.80 ± 3.55 ^d^	0 ± 0 ^i^

Means ± SD followed by the same letter in each column are not significantly different at *p* < 0.05 when analyzed using Duncan’s test of one-way ANOVA.

**Table 2:** Effects of *Aspergillus unguis* KUFA 0098 on the growth of the ten plant pathogenic fungi.

Plant Pathogen	MIC (µg/mL)			
	1	2	4	Carbendazim
*Alternaria brassicicola*	125	>500	500	125
*Bipolaris oryzae*	125	>500	>500	62.5
*Colletotrichum capsici*	125	>500	125	125
*Curvularia oryzae*	125	>500	>500	125
*Fusarium semitectum*	250	>500	250	125
*Lasiodiplodia theobromae*	>500	>500	>500	250
*Phytophthora palmivora*	125	>500	125	62.5
*Pyricularia oryzae*	125	>500	125	125
*Rhizoctonia oryzae*	>500	>500	>500	250
*Sclerotium roflsii*	>500	>500	>500	250

## Data Availability

The data presented in this study are available on request from the corresponding author.

## Supplementary Materials

The following are available online at https://www.mdpi.com/article/10.3390/md23120461/s1. Figures S1–S25: 1D and 2D NMR spectra of compounds **1**–**5**. Figures S26 and S27: HRMS data for compounds **2** and **3**. Figure S28: The design of rice seedling pot arrangement in the greenhouse. Tables S1–S5: ^1^H and ^13^C NMR data of compounds **1**–**5**.
