# Supplementary material for: In Vitro and In Vivo Anti-Phytopathogenic Fungal Activity of a Culture Extract of the Marine-Derived Fungus, Aspergillus unguis KUFA 0098, and Its Major Depsidone Constituents
Source: Mar Drugs. 2025 Nov 29;23(12):461. doi: 10.3390/md23120461 (PMC12734577; doi:10.3390/md23120461)

Supplementary Information (SI)

In Vitro and *In Vivo* Anti-phytopathogenic Fungal Activity of a Culture Extract of the Marine-Derived Fungus, *Aspergillus unguis* KUFA0098, and its Major Depsidone Constituents

Decha Kumla [1†], Diana I. C. Pinho [2,7†], Emília Sousa [2], Tida Dethoup [3*], Luis Gales [4], Sharad Mistry [5], Artur M.S. Silva [6], Anake Kijjoa [7*]

Table S1 ^{1}H and ^{13}C NMR (DMSO-d6, 300 MHz and 75 MHz) and HMBC assignment for unguinol (**1**).

Position	δC, type	δH (*J* in Hz)	COSY	HMBC
1	145.0 C	-		
2	116.1CH	6.56 dd (2.3, 0.7)	H-4, 12	C-4, 11a. 12
3	162.9 C	-		
4	104.9 CH	6.33 d (2.3)	H-2, 12	C-2, 3, 4a, 11a
4a	163.5 C	-		
5a	140.8 C	-		
6	135.6 C	-		
7	111.1 CH	6.45s	H-13	C-5a, 6, 8, 9, 13
8	153.0 C	-		
9	115.1 C	-		
9a	143.7 C	-		
11	162.3 CO	-		
11a	112.0 C	-		
12	21.1 CH_3	2.34s	H-2, 4	C-1, 2, 11a
13	9.7 CH_3	2.07s	H-7	C-8, 9, 9a
1’	132.7 C	-		
2’	125.6 CH	5.51 m	H-3’, 4’	C-1’, 3’, 4’
3’	18.0 CH_3	2.01 m	H-2’, 4’	C-C-1’, 2’, 6
4’	14.1 CH_3	1.81 brd (6.8)	H-2’, 3’	C-2’, 6’

Table S2 ^{1}H and ^{13}C NMR (DMSO-d6, 300 MHz and 75 MHz) and HMBC assignment for 2-chlorounguinol (**2**).

Position	δC, type	δH (*J* in Hz)	COSY	HMBC
1	141.4 C	-		
2	119.5 C			
3	157.9 C			
4	105.4 CH	6.60 d (0.2)	H-12	C-1, 3, 4, 11a
4a	161.2 C			
5a	140.7 C			
6	135.7 C			
7	111.2 CH	6.46 s	H-13	C-1’, 5a, 8, 9, 13
8	153.1 C			
9	115.2 C			
9a	143.5 C			
11	162.7 CO			
11a	113.8 C			
12	18.5 CH_3	2.38 s		C-1, 2, 3, 4, 4a, 11a
13	9.6 CH_3	2.06 s		C-8, 9, 9a
1’	132.4 C			
2’	125.8 CH	5.51 m	H-3’, 4’	C-3’, 4’, 6
3’	14.3 CH_3	1.82 brd (6.8)	H-4’	C-1’, 2’
4’	18.0 CH_3	1.96 m	H-2, 3’	C-1’, 2’, 6
OH-3	-	11.43 br		C-9
OH-8	-	9.67 brs		

Table S3 ^{1}H and ^{13}C NMR (DMSO-d6, 300 MHz and 75 MHz) and HMBC assignment for 2, 4-dichlorounguinol (**3**).

Position	δC, type	δH (*J* in Hz)	COSY	HMBC
1	139.1 C	-		
2	115.3 C	-		
3	154.0 C	-		
4	111.4 C	-		
4a	157.4 C	-		
5a	141.3 C	-		
6	136.4C	-		
7	111.8 CH	6.45 s		C-1', 5a, 8, 9, 13
8	153.5 C	-		
9	115.3 C	-		
9a	143.1 C	-		
11	162.2 CO	-		
11a	120.9 C	-		
12	18.9 CH_3	2.39 s		C-1, 2, 11a
13	9.7 CH_3	2.08 s		C-8, 9, 9a
1'	134.0 C	-		
2'	125.4 CH	5.43 m	C-3'	C-3', 4', 6
3'	14.4 CH_3	1.73 brd (7.0)	C-2'	C-1', 2'
4'	18.4 CH_3	2.02 m		C-1', 2', 6
OH-8	-	9.80 brs		C-8, 9

Table S4 ^{1}H and ^{13}C NMR (DMSO-d6, 300 MHz and 75 MHz) and HMBC assignment for folipastatin (**4**).

Position	δC, type	δH (*J* in Hz)	COSY	NOESY	HMBC
1	136.0 C	-			
2	112.3 CH	6.56s		H-2', 4'	C-1, 3, 11, 11a, 12
3	159.9 C	-			
4	113.9 C	-			
4a	162.1 C	-			
5a	142.2 C	-			
6	136.2 C	-			
7	111.5 CH	6.43 s		H-2", 4"	C-1", 5[a], 8, 9, 13
8	153.0 C	-			
9	115.0 C	-			
9a	143.7 C	-			
11	164.1 CO	-			
11a	111.4 C	-			
12	8.8 CH_3	2.06 s			C-3, 4, 4a
13	9.5 CH_3	2.07 s			C-8, 9, 9a
1'	148.1 C	-			
2'	124.3 CH	5.35 m	H-3', 4'	H-2, 3'	C-1', 3', 4'
3'	14.5 CH_3	1.67 dd (6.8, 1.0)	H-2'	H-2'	C-C-1, 2'
4'	17.9 CH_3	1.80 brt (1.0)			C-1, 1', 2'
1"	134.1 C	-			
2"	125.6 CH	5.49 m	H-3", 4"	H-7, 3"	C-3", 4", 6
3"	14.3 CH_3	1.74 brd (6.8)	H-2"	H-2"	C-1", 2"
4"	18.3 CH_3	1.99 m			C-1", 2", 6
OH-3	-	10.44 brs			
OH-7	-	9.73 brs			

Table S5 ^{1}H and ^{13}C NMR (DMSO-d6, 300 MHz and 75 MHz) and HMBC assignment for aspergillusphenol A (**5**).

Position	δ_C, type	δ_H (J in Hz)	COSY	HMBC
1	156.3, C	-		
2	109.1C	-		
3	156.3 C	-		
4	103.7 CH	6.38 s		C-1,2,3,6,7
5	141.1 C	-		
6	103.7, CH	6.38 s		C-1,2,3,4,6,7
7	8.9 CH_3	1.91 s		C-1, 2,3,4,6
8	135.5 C	-		
9	120.5 CH	5.70 m		C-5,10
10	14.5 CH_3	1.72 brd (6.8)	H-9, 11	C-8, 9
11	15.7 CH_3	1.87 m		C-1, 8, 9
OH-1, 3	-	8.98s		C-4,6

Fig. S1 ^{1}H NMR spectrum of unguinol (**1**) (DMSO-d6, 300 MHz).

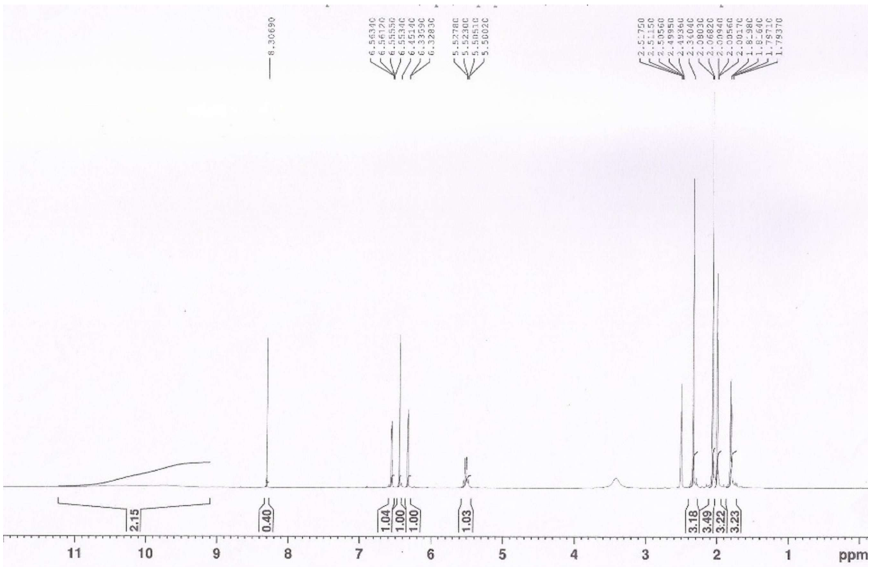

Fig. S2 ^{13}C NMR spectrum of unguinol (**1**) (DMSO-d6, 75 MHz).

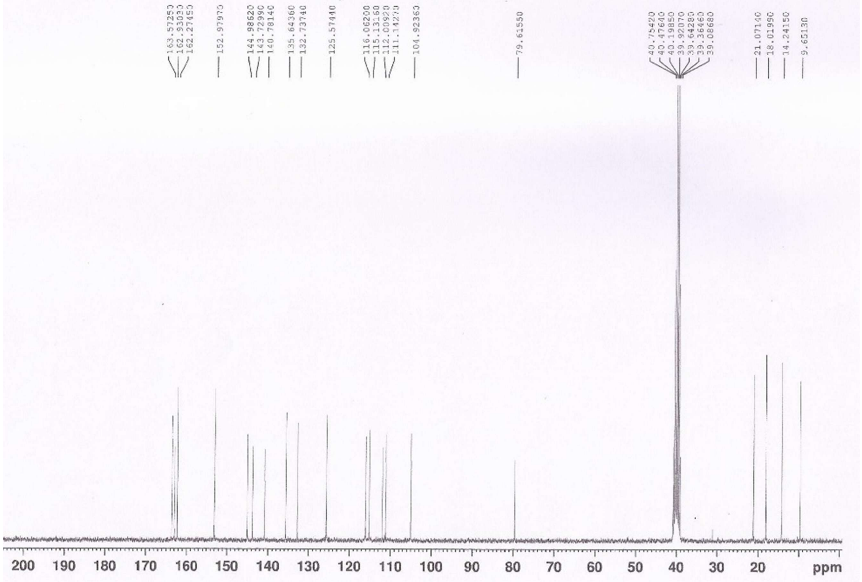

Fig S3. COSY spectrum of unguinol (**1**) (DMSO-d6, 300 MHz).

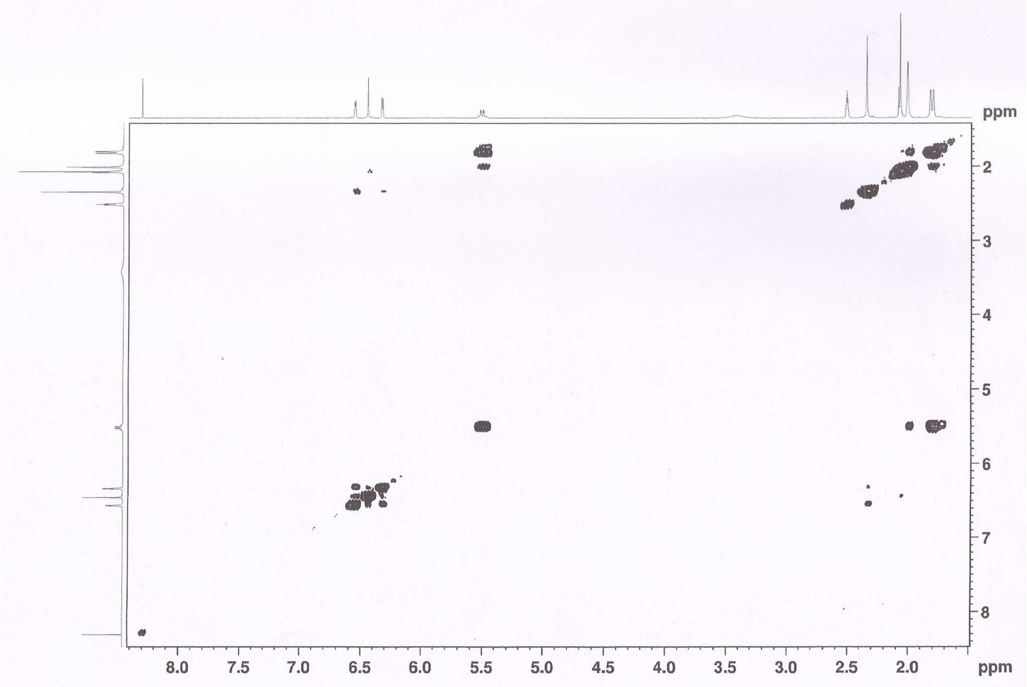

Fig. S4 HSQC spectrum of unguinol (**1**) (DMSO-d6, 300 MHz).

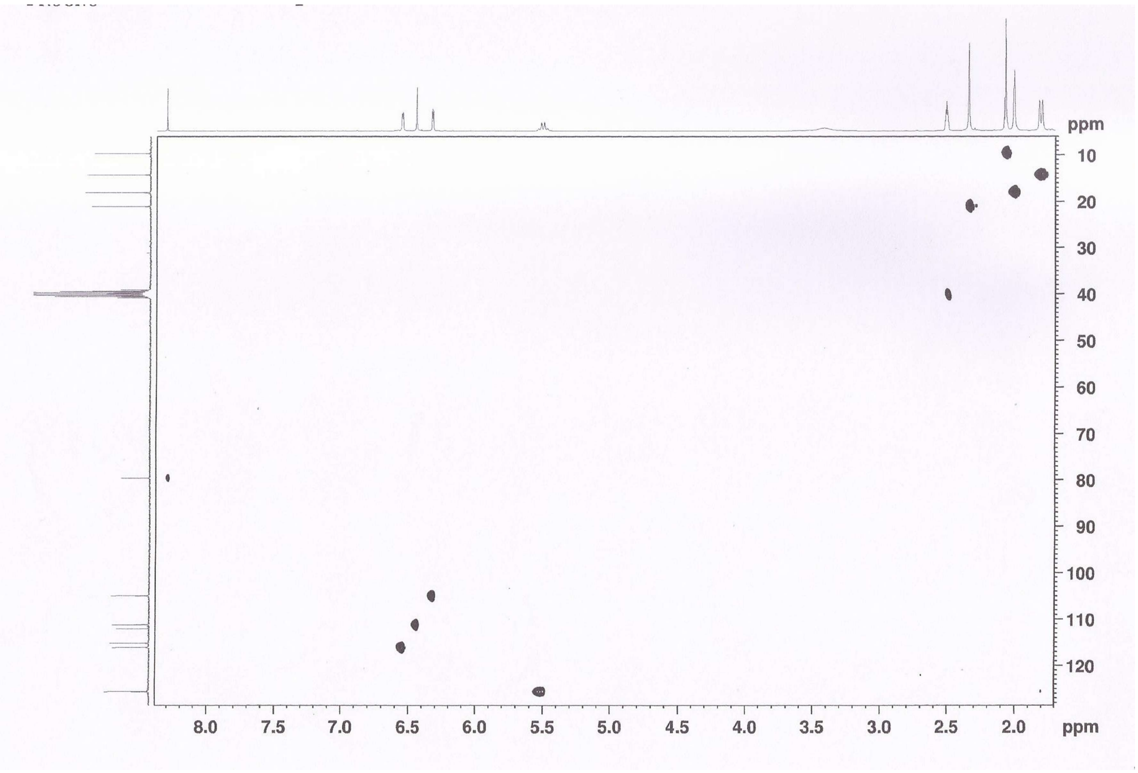

Fig. S5 HMBC spectrum of unguinol (**1**) (DMSO-d6, 300 MHz).

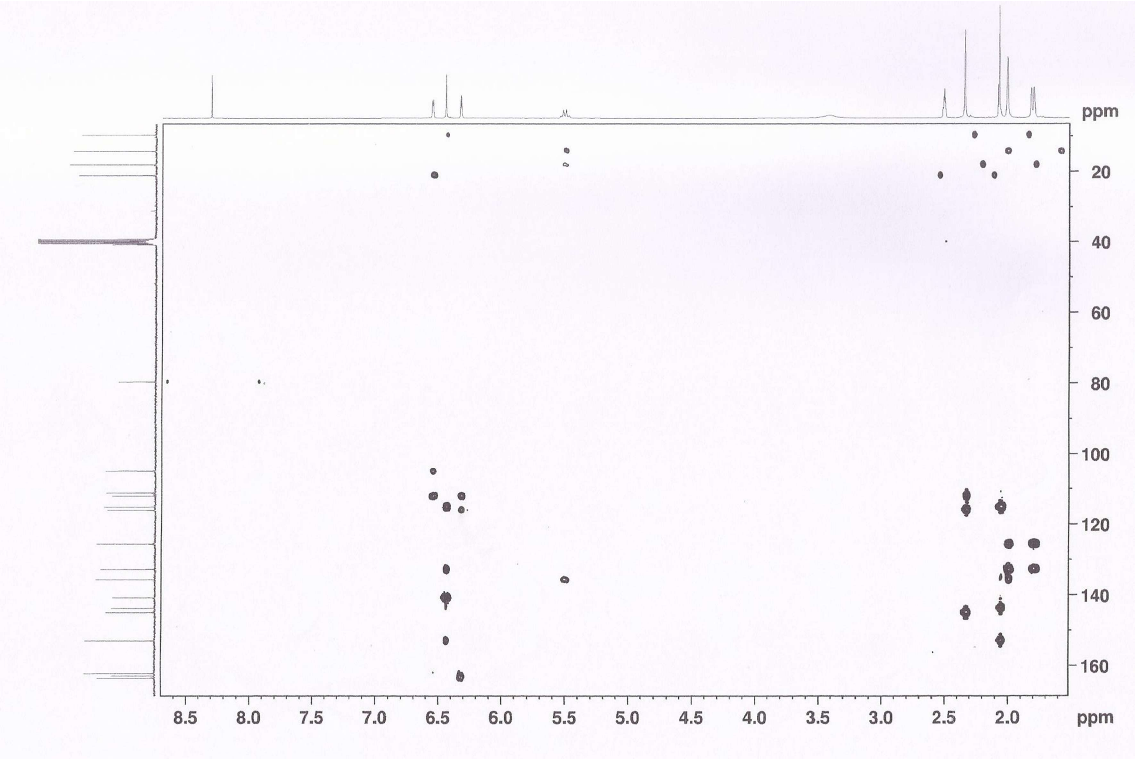

Fig. S6 ^{1}H NMR spectrum of 2-chlorounguinol (**2**) (DMSO-d6, 300 MHz).

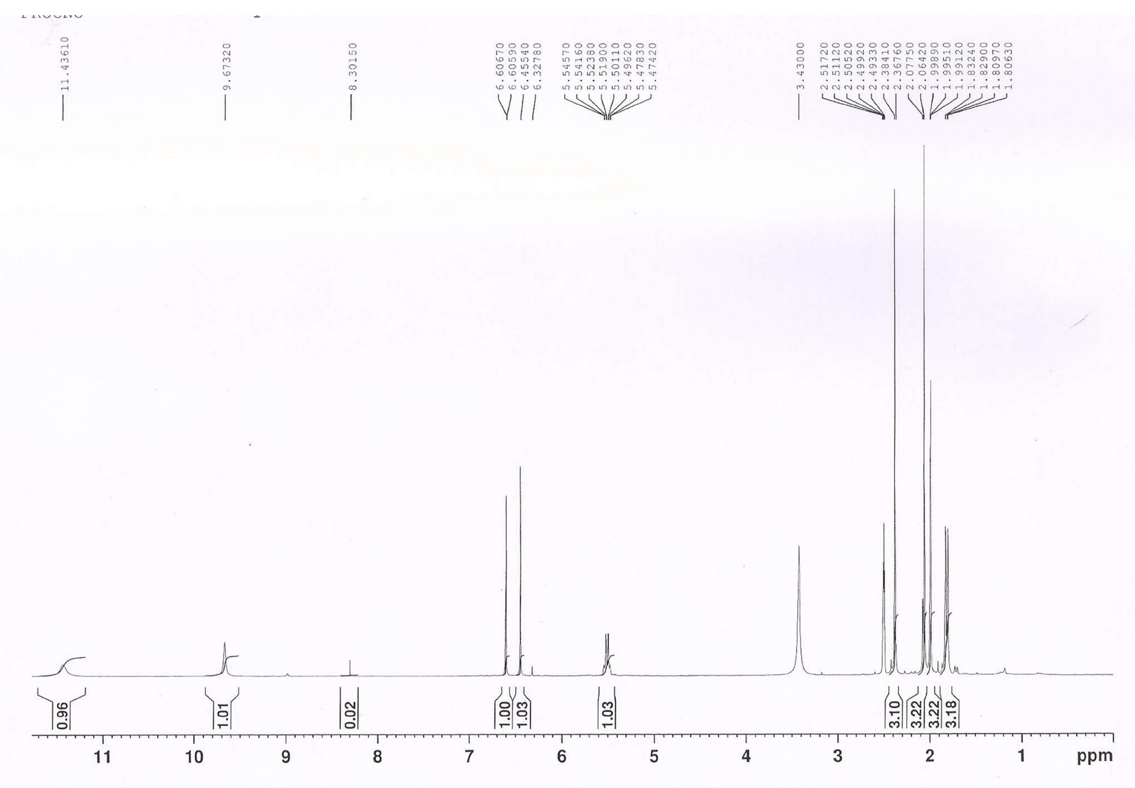

Fig. S7 ^{13}C NMR spectrumof 2-chlorounguinol (**2**) (DMSO-d6, 75 MHz).

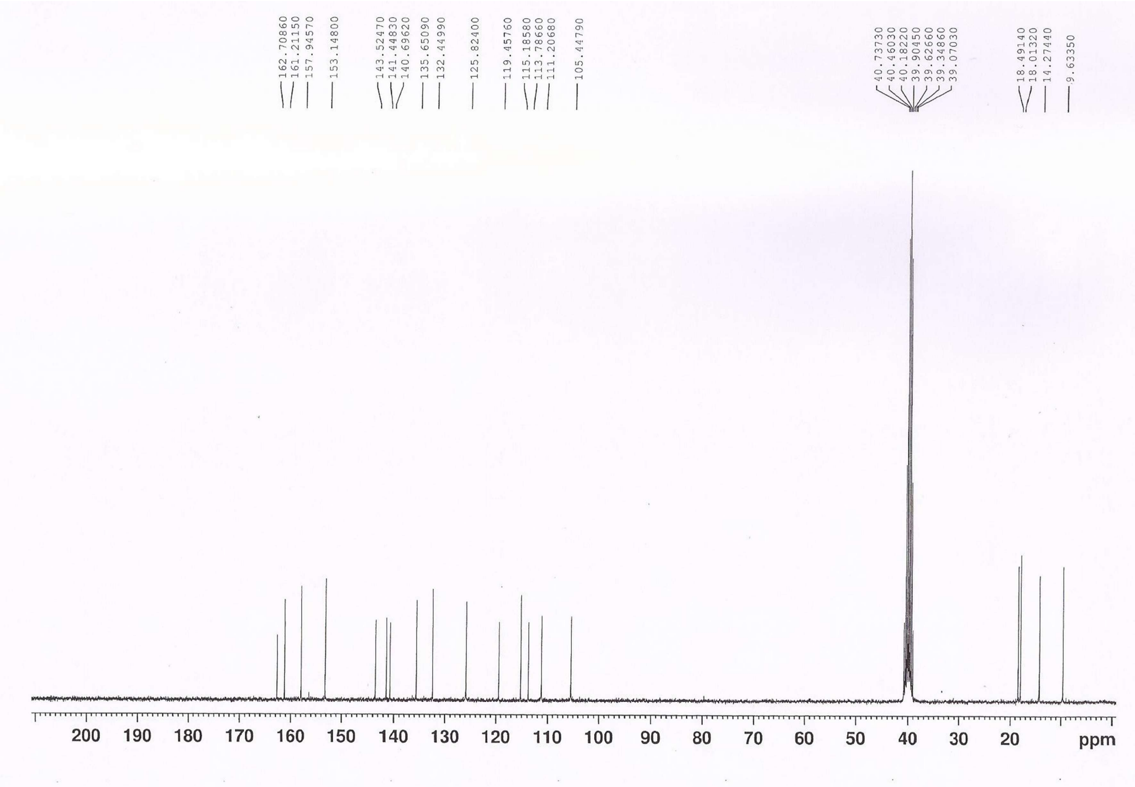

Fig. S8 COSY spectrum of 2-chlorounguinol (**2**) (DMSO-d6, 300 MHz).

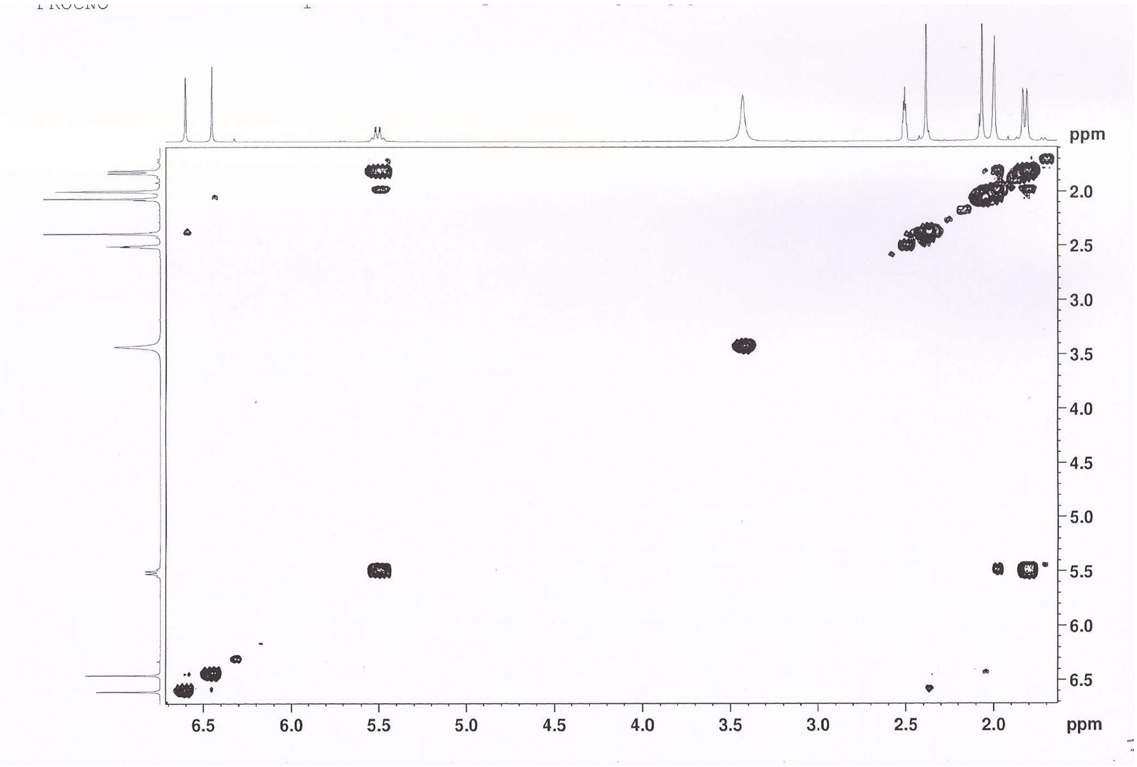

Fig. S9 HSQC sepectrum of 2-chlorounguinol (**2**) (DMSO-d6, 300 MHz).

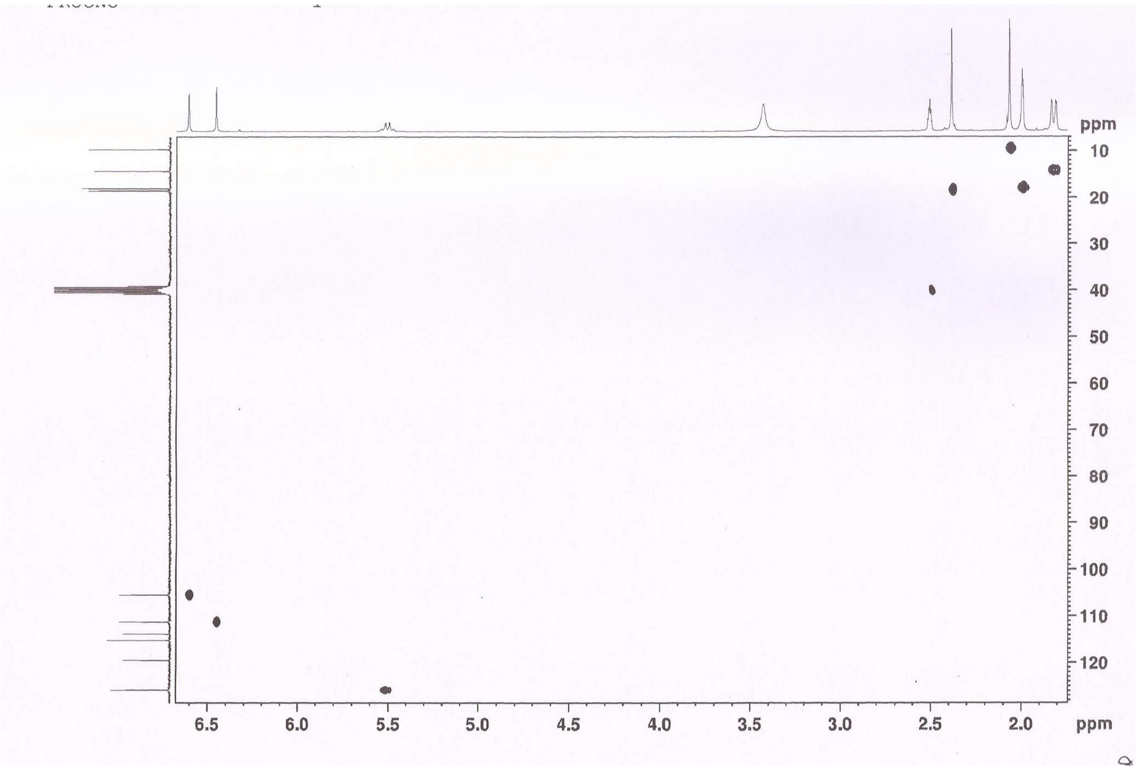

Fig. S10 HMBC sepectrum of 2-chlorounguinol (**2**) (DMSO-d6, 300 MHz).

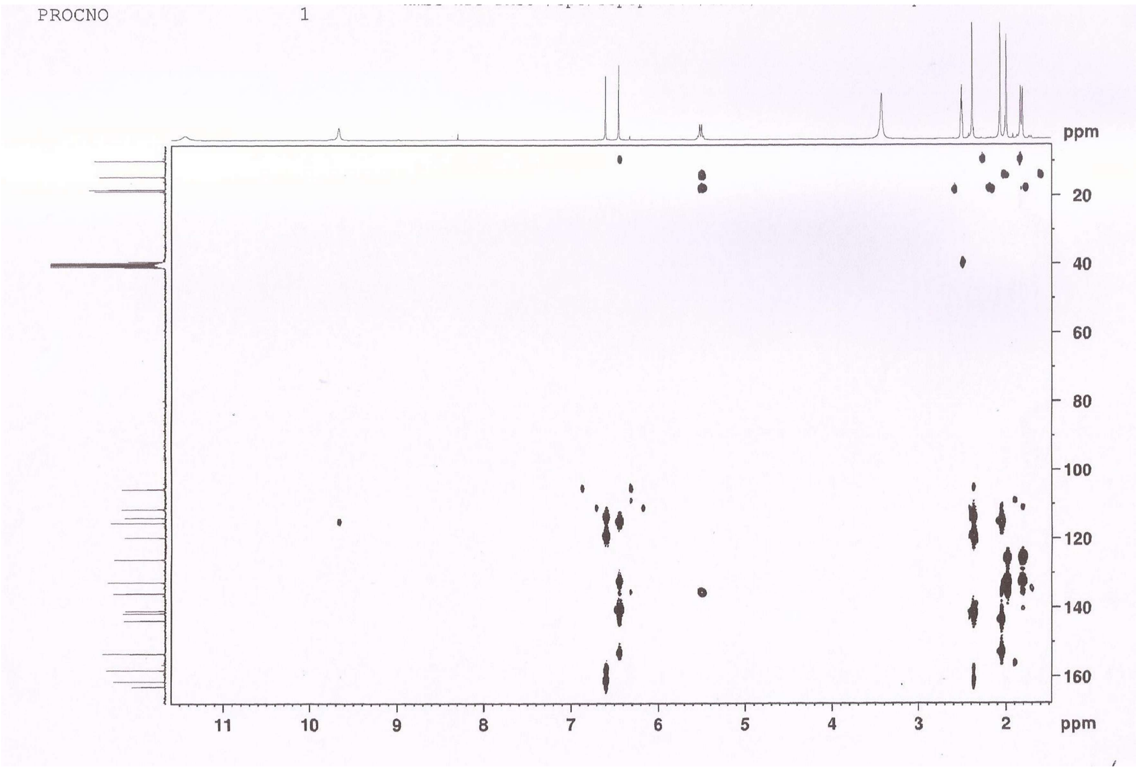

Fig. S11 ^{1}H NMR spectrum of 2,4-dichlorounguinol (**3**) (DMSO-d6, 300 MHz).

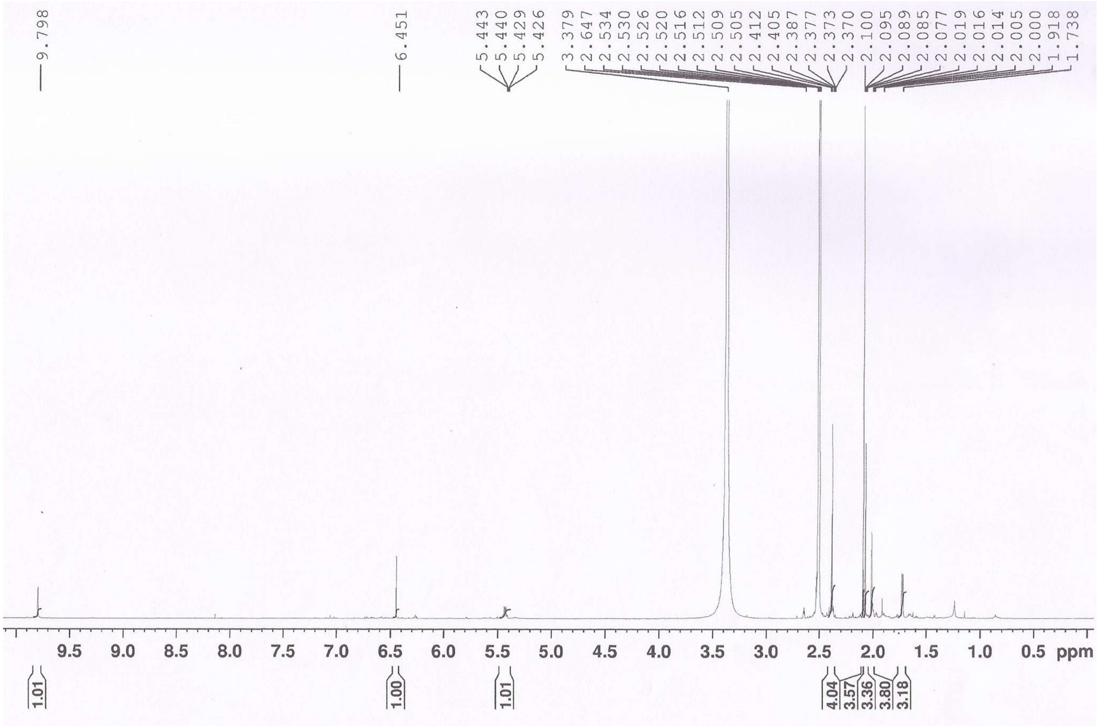

Fig. S12. ^{13}C NMR spectrumof 2,4-dichlorounguinol (**3**) (DMSO-d6, 75 MHz).

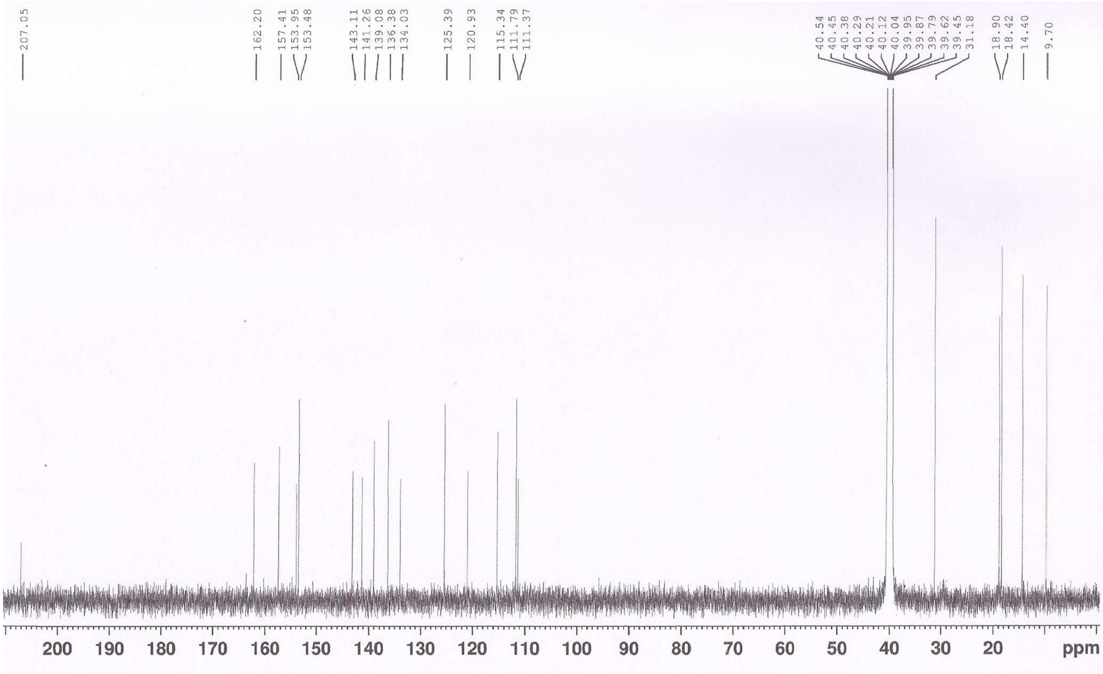

Fig. S13 COSY spectrum of 2,4-dichlorounguinol (**3**) (DMSO-d6, 300 MHz).

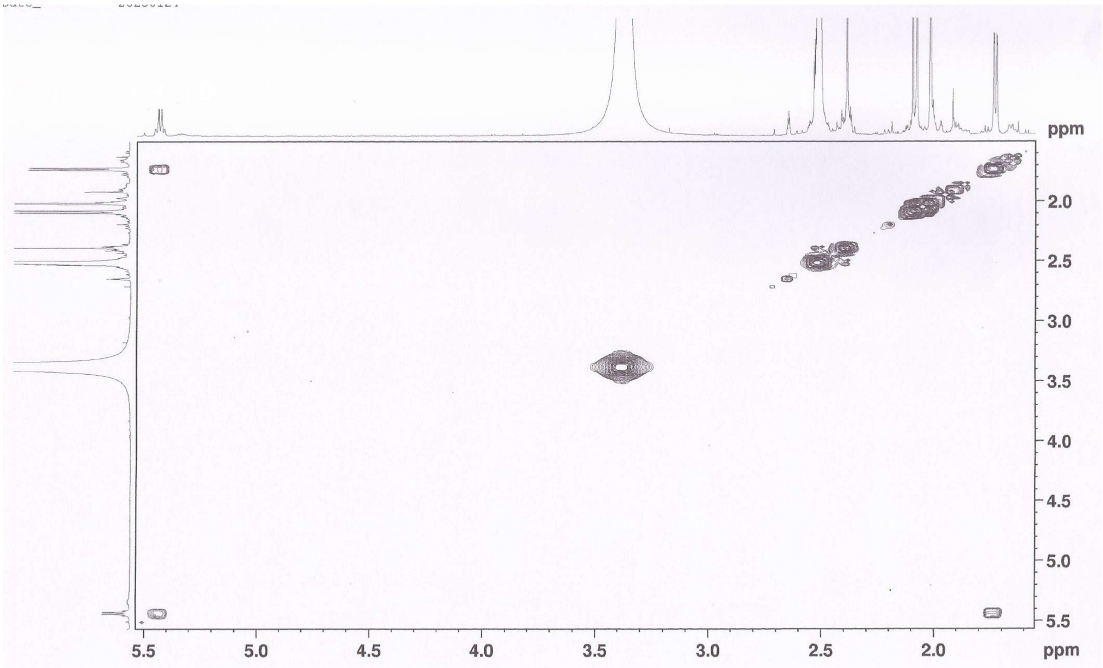

Fig. S14 HSQC sepectrum of 2,4-dichlorounguinol (**3**) (DMSO-d6, 300 MHz).

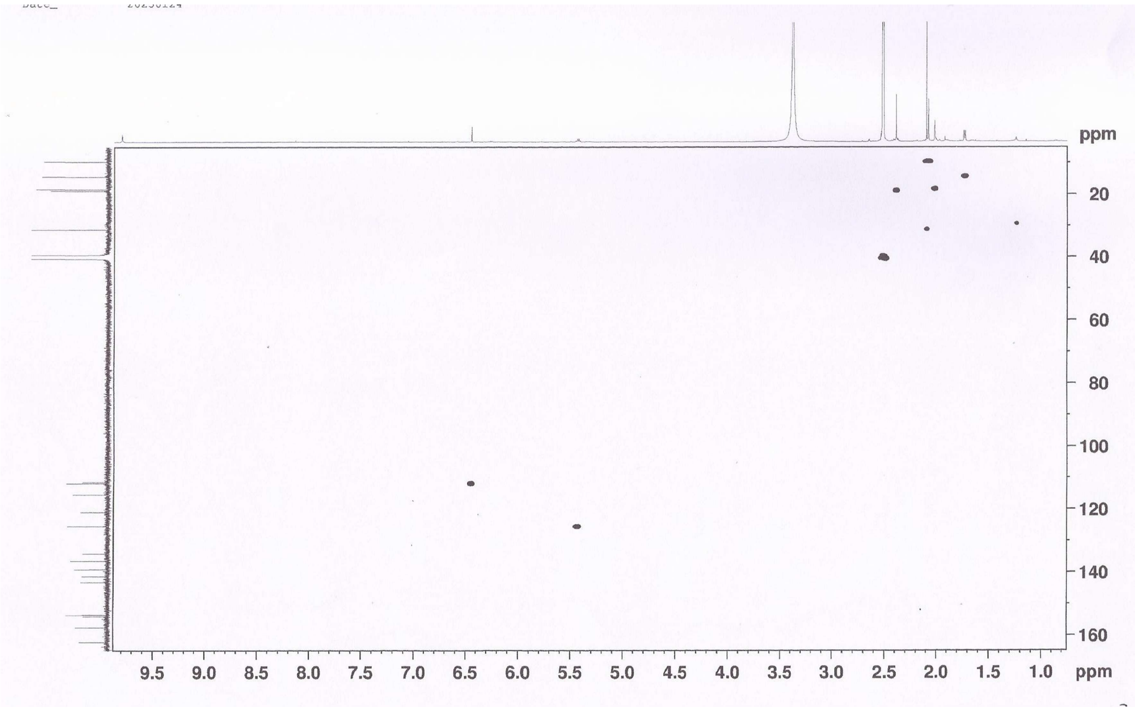

Fig. S15 HMBC sepectrum of 2,4-dichlorounguinol (3) (DMSO-d6, 300 MHz).

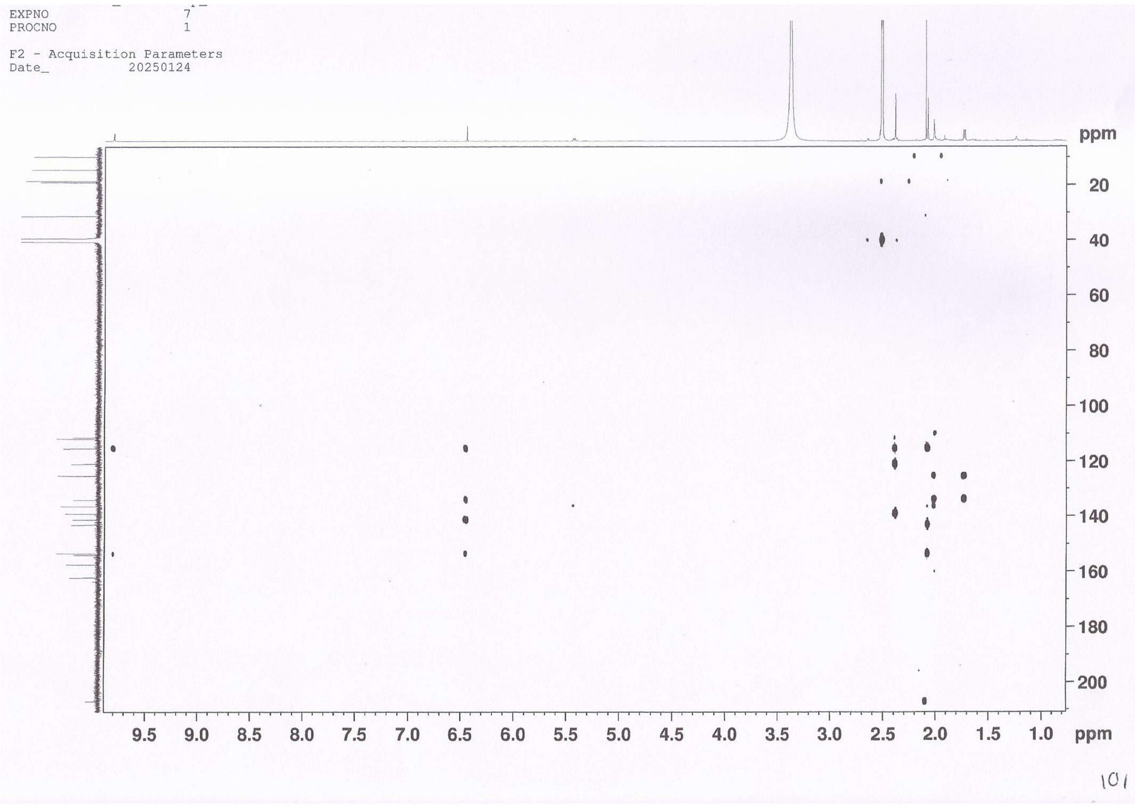

Fig. S16 ^{1}H NMR spectrum of folipastatin (**4**) (DMSO-d6, 300 MHz).

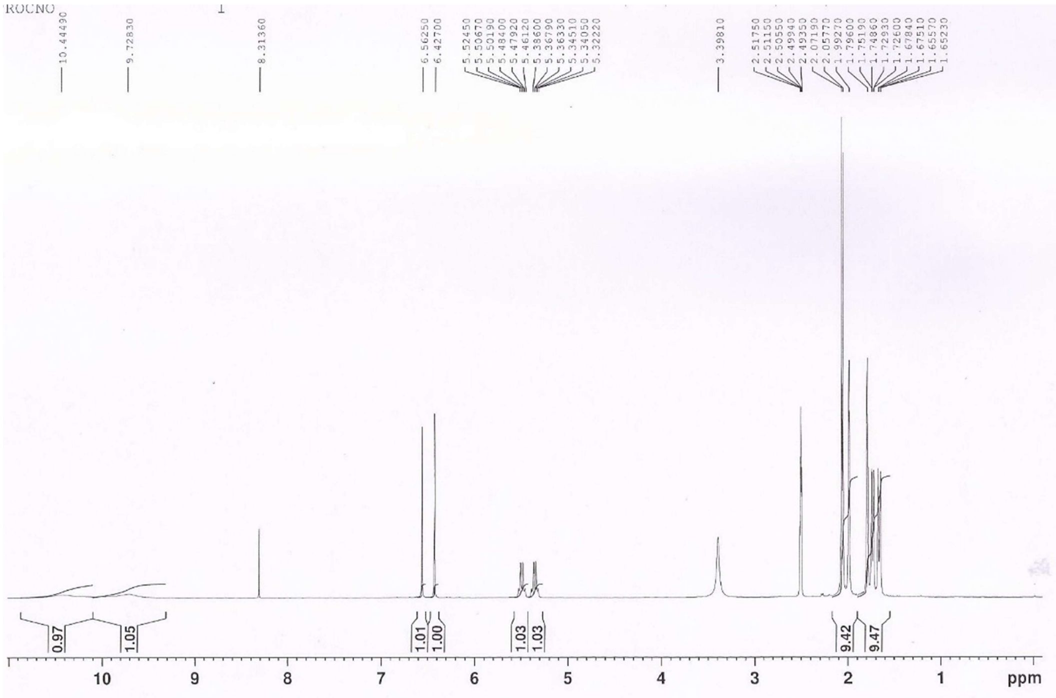

Fig. S17 ^{13}C NMR spectrumof folipastatin (**4**) (DMSO-d6, 75 MHz).

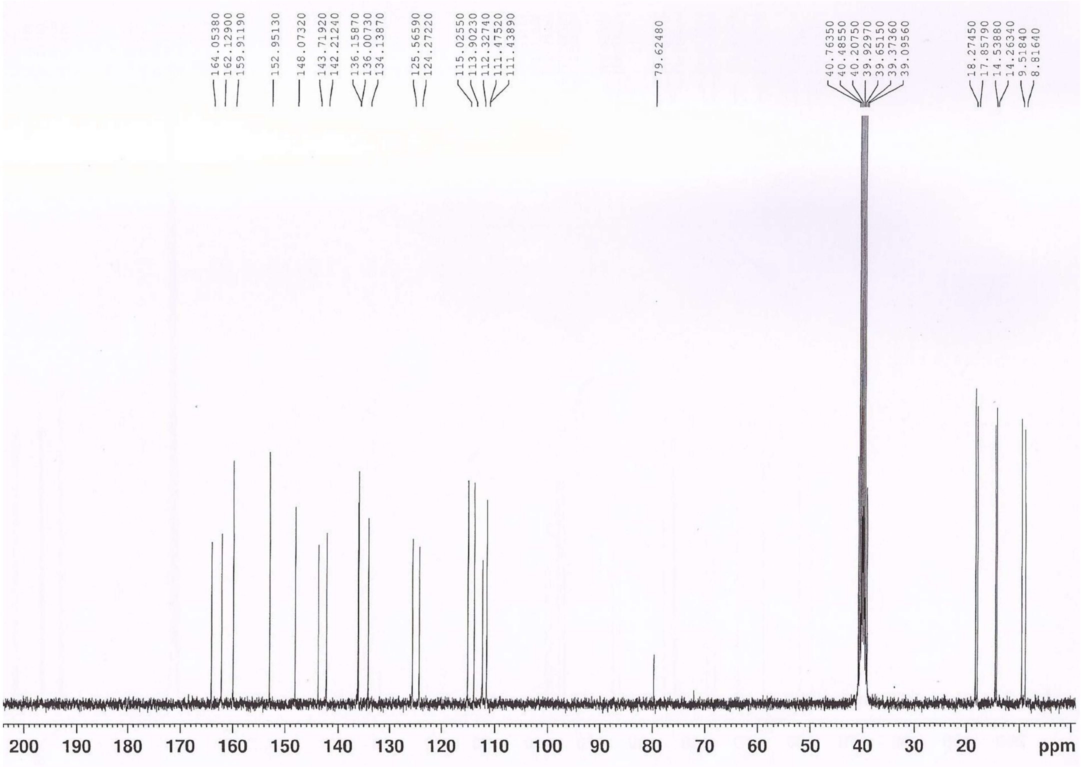

Fig. S18 COSY spectrum of folipastatin (**4**) (DMSO-d6, 300 MHz).

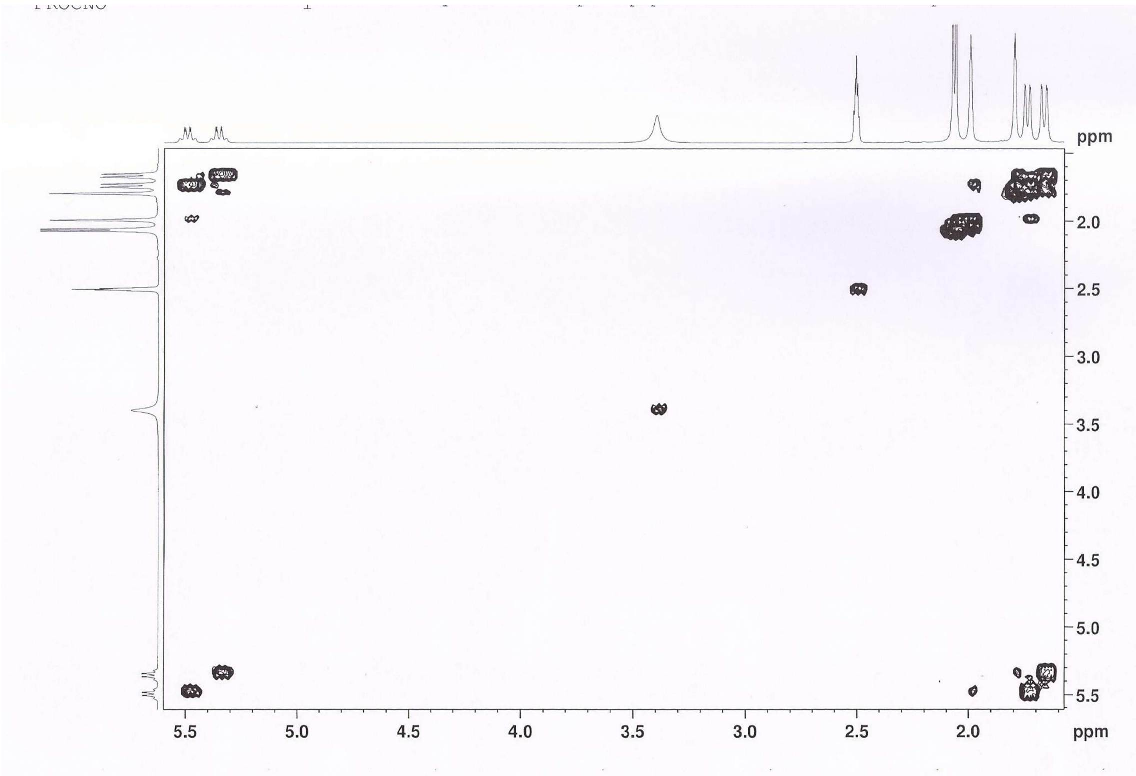

Fig. S19 HSQC sepectrum of folipastatin (**4**) (DMSO-d6, 300 MHz).

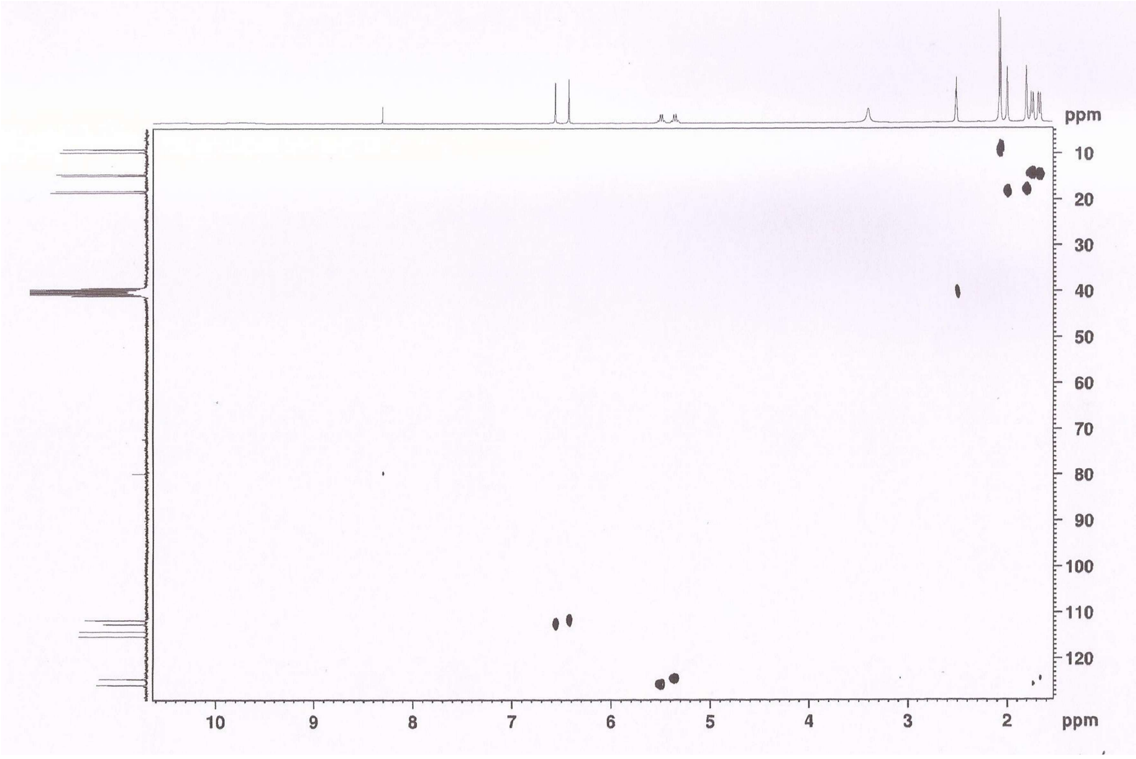

Fig. S20 HMBC sepectrum of folipastatin (**4**) (DMSO-d6, 300 MHz).

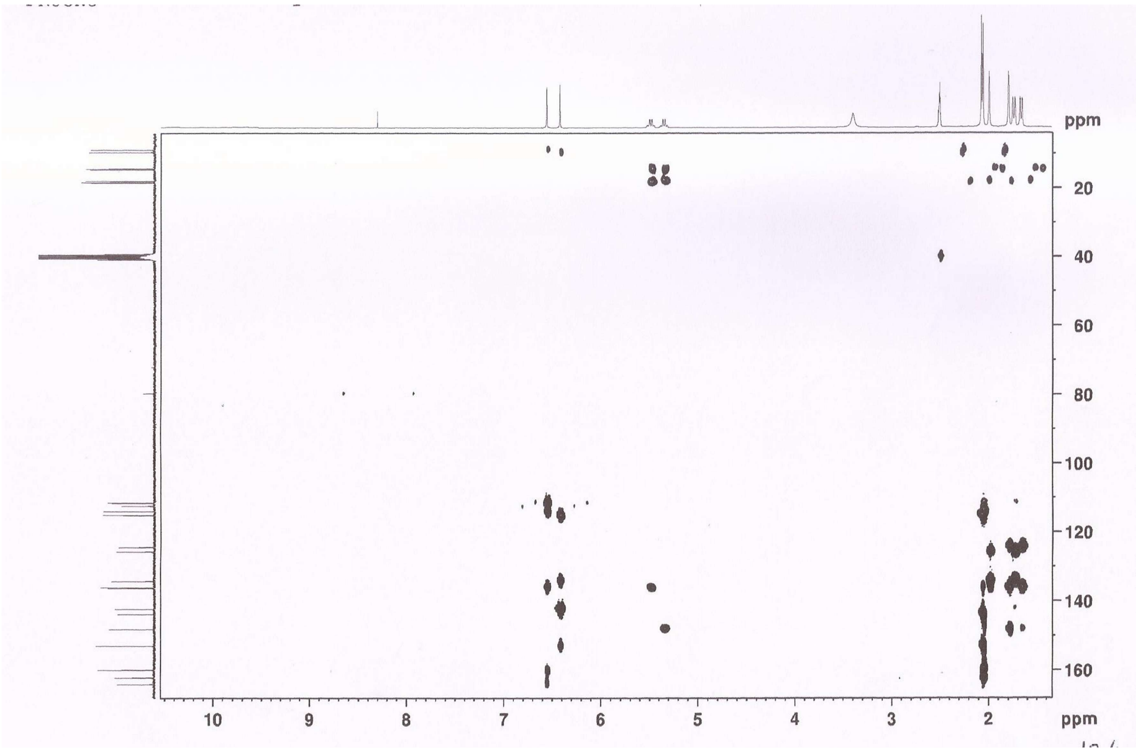

Fig. S21 ^{1}H NMR spectrum of aspergillusphenol A (**5**) (DMSO-d6, 300 MHz).

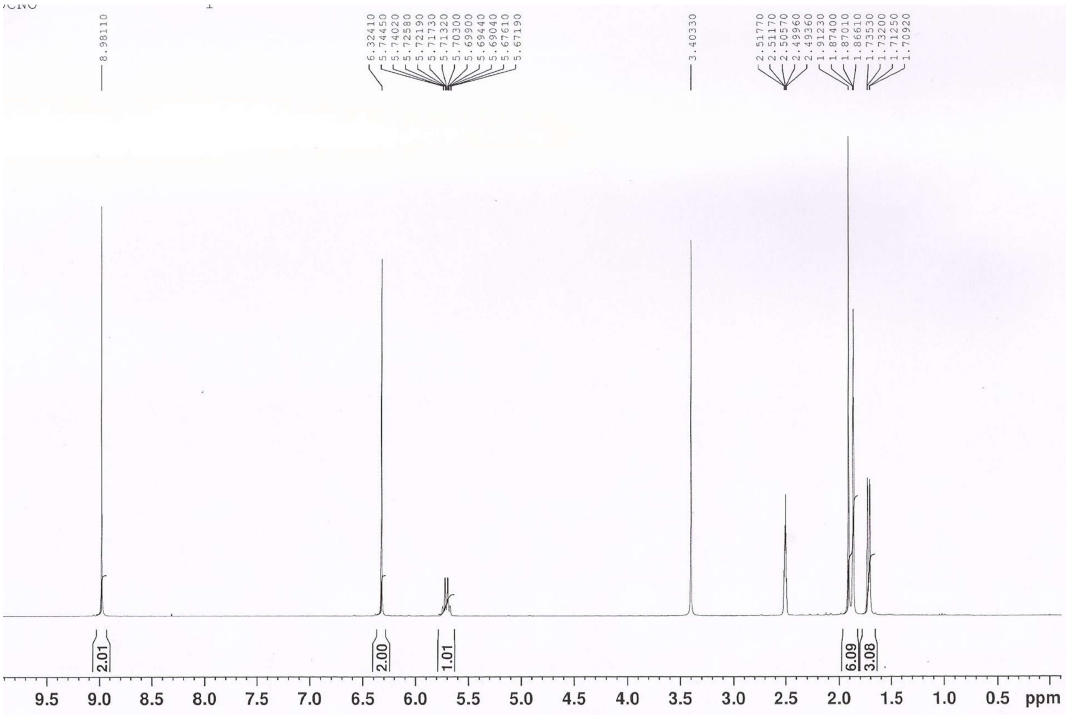

Fig. S22 ^{13}C NMR spectrumof aspergillusphenol A (**5**) (DMSO-d6, 75 MHz).

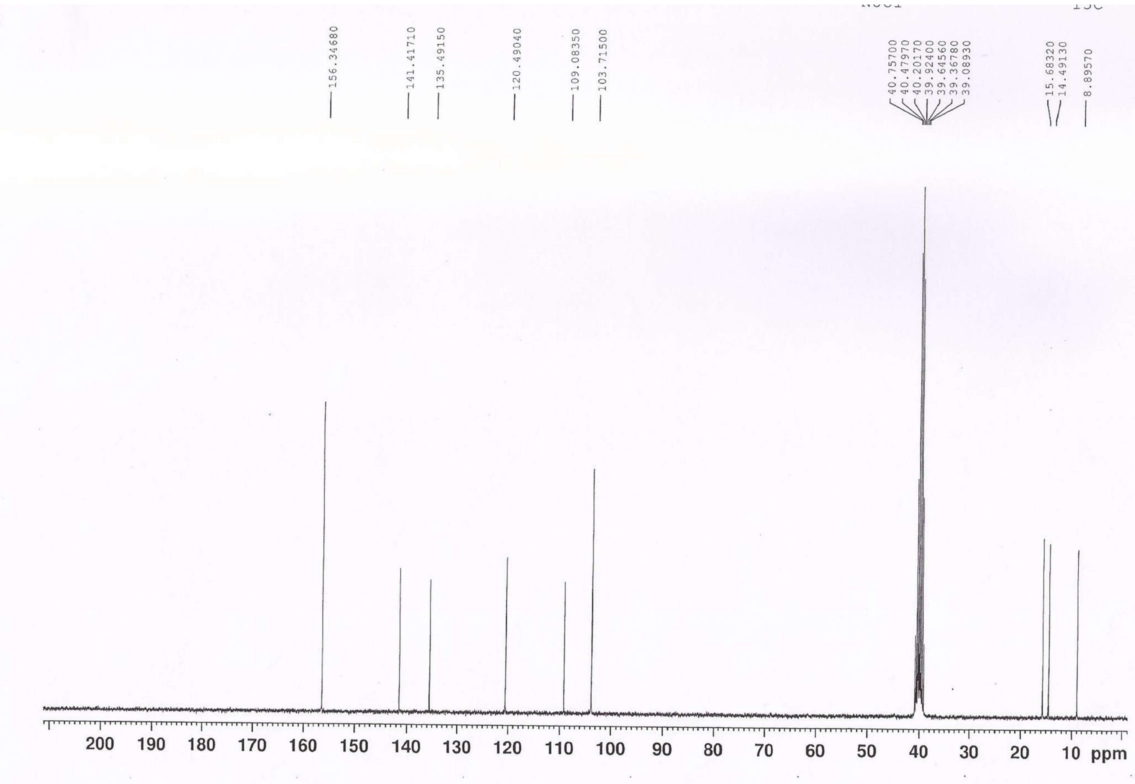

Fig. S23 COSY spectrum of aspergillusphenol A (**5**) (DMSO-d6, 300 MHz).

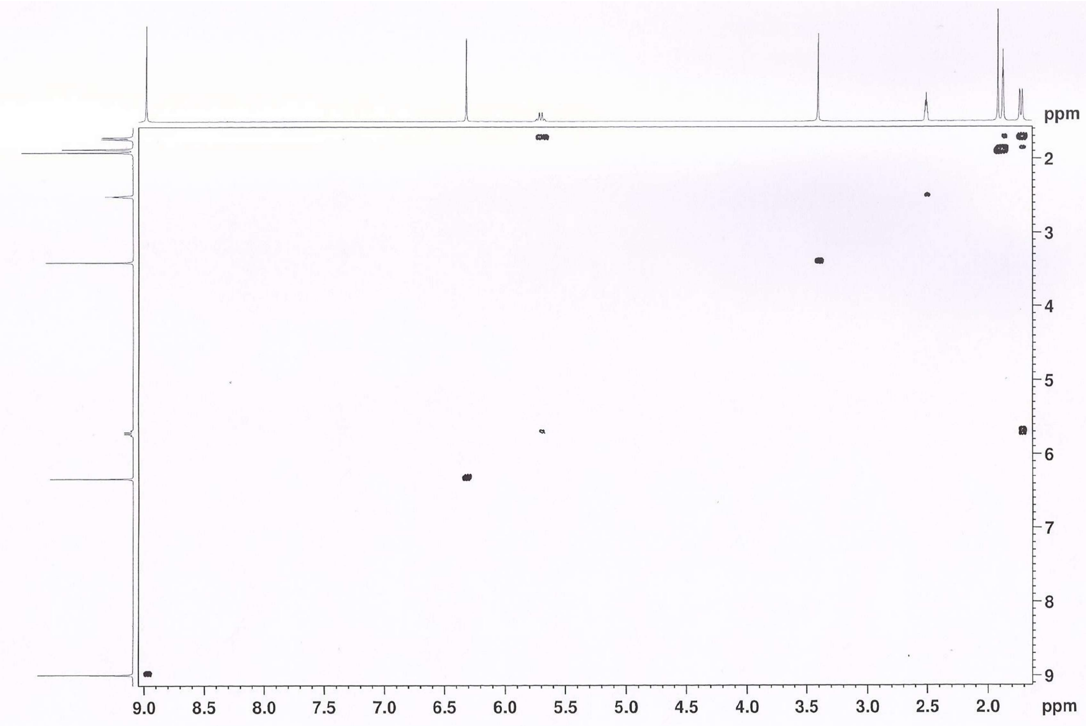

Fig. S24 HSQC sepectrum of aspergillusphenol A (**5**) (DMSO-d6, 300 MHz).

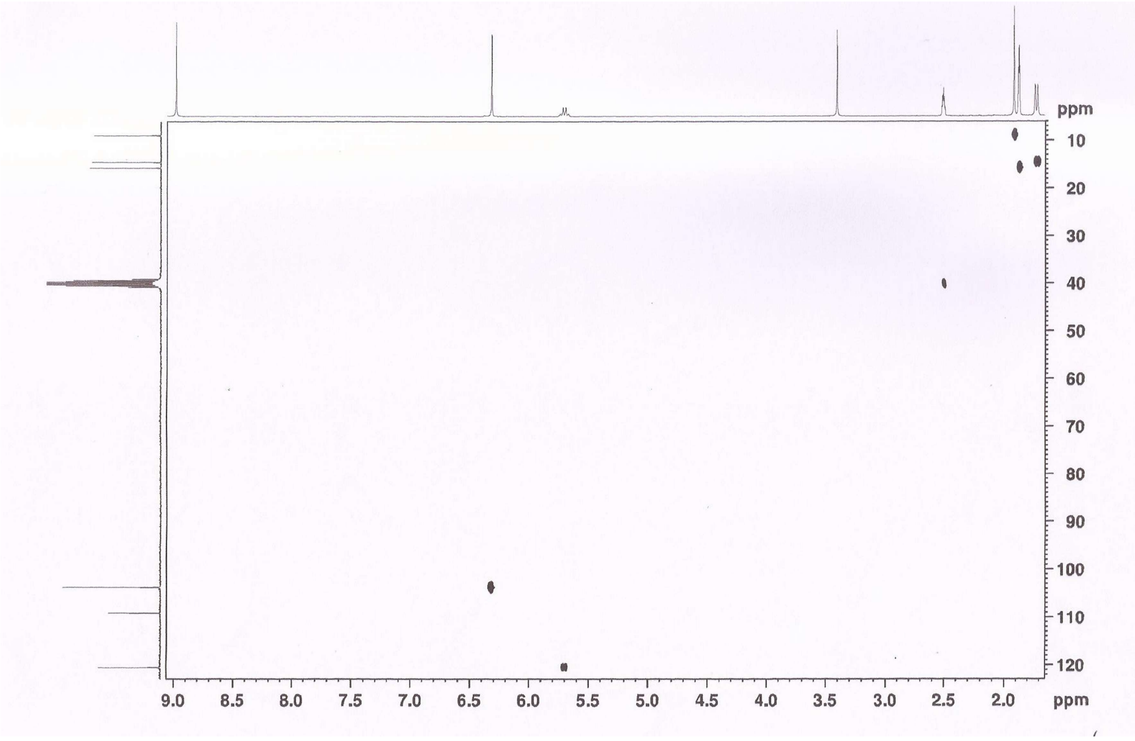

Fig. S25 HMBC sepectrum of aspergillusphenol A (**5**) (DMSO-d6, 300 MHz).

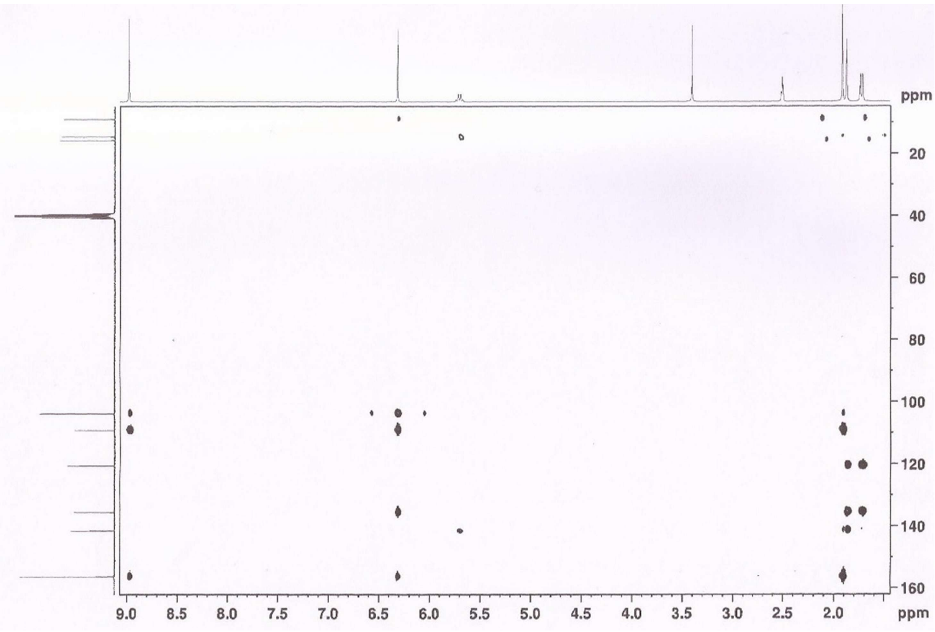

Fig. S26 (+)-HRESIMS of 2-chlorounguinol (**2**).

Elemental Composition Report $[MH]^+$ 35Cl
Single Mass Analysis
Tolerance = 5.0 PPM / DBE: min = 1.5, max = 100.0
Element prediction: Off
Number of isotope peaks used for i FIT = 3
Monoisotopic Mass, Even Electron Ions
523 formula(e) evaluated with 1 results within limits (all results (up to 1000) for each mass)
Elements Used:
C: 19 19 H: 0 150 O: 0 30 35Cl: 0 8 37Cl: 0 8

Minimum:				1.5				
Maximum:		5.0	5.0	100.0				
Mass	Calc. Mass	mDa	PPM	DBE	i FIT	Norm	Conf(%)	Formula
361.0848	361.0843	0.5	1.4	10.5	498.0	n/a	n/a	C19 H18 O5 35Cl

Elemental Composition Report $[MH]^+$ 37Cl
Single Mass Analysis
Tolerance = 5.0 PPM / DBE: min = 1.5, max = 100.0
Element prediction: Off
Number of isotope peaks used for i FIT = 3
Monoisotopic Mass, Even Electron Ions
529 formula(e) evaluated with 1 results within limits (all results (up to 1000) for each mass)
Elements Used:
C: 19 19 H: 0 150 O: 0 30 35Cl: 0 8 37Cl: 0 8

Minimum:				1.5				
Maximum:		5.0	5.0	100.0				
Mass	Calc. Mass	mDa	PPM	DBE	i FIT	Norm	Conf(%)	Formula
363.0828	363.0813	1.5	4.1	10.5	726.0	n/a	n/a	C19 H18 O5 37Cl

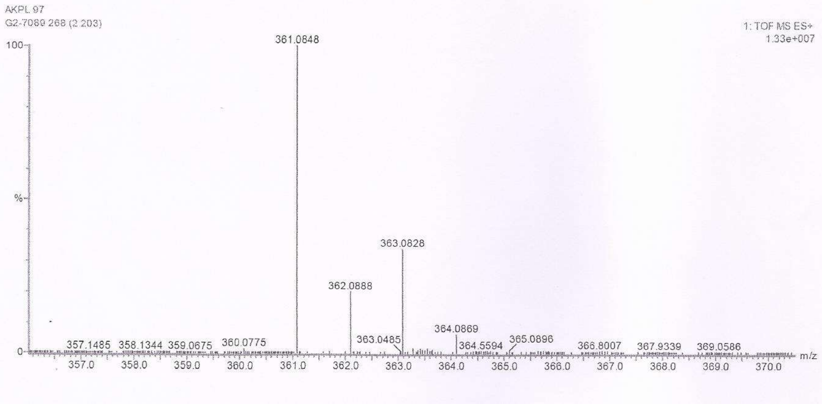

Fig. S27 (+)-HRESIMS of 2,4-dichlorounguinol (**3**).

Elemental Composition Report [MH]+ 2 x 35Cl
Single Mass Analysis
Tolerance = 5.0 PPM / DBE: min = -1.5, max = 100.0
Element prediction: Off
Number of isotope peaks used for i-FIT = 3
Monoisotopic Mass, Even Electron Ions
652 formula(e) evaluated with 1 results within limits (all results (up to 1000) for each mass)
Elements Used:
C: 19-19 H: 0-1050 O: 0-30 35Cl: 0-8 37Cl: 0-8

Minimum:				-1.5				
Maximum:		5.0	5.0	100.0				
Mass	Calc. Mass	mDa	PPM	DBE	i-FIT	Norm	Conf(%)	Formula
395.0450	395.0453	-0.3	-0.8	10.5	1227.0	n/a	n/a	C19 H17 O5 35Cl2

Elemental Composition Report [MH]+ 1 x 35Cl, 1 x 37Cl
Single Mass Analysis
Tolerance = 5.0 PPM / DBE: min = -1.5, max = 100.0
Element prediction: Off
Number of isotope peaks used for i-FIT = 3
Monoisotopic Mass, Even Electron Ions
659 formula(e) evaluated with 1 results within limits (all results (up to 1000) for each mass)
Elements Used:
C: 19-19 H: 0-1050 O: 0-30 35Cl: 0-8 37Cl: 0-8

Minimum:				-1.5				
Maximum:		5.0	5.0	100.0				
Mass	Calc. Mass	mDa	PPM	DBE	i-FIT	Norm	Conf(%)	Formula
397.0426	397.0424	0.2	0.5	10.5	1203.0	n/a	n/a	C19 H17 O5 35Cl 37Cl

Elemental Composition Report [MH]+ 2 x 37Cl
Single Mass Analysis
Tolerance = 5.0 PPM / DBE: min = -1.5, max = 100.0
Element prediction: Off
Number of isotope peaks used for i-FIT = 3
Monoisotopic Mass, Even Electron Ions
667 formula(e) evaluated with 1 results within limits (all results (up to 1000) for each mass)
Elements Used:
C: 19-19 H: 0-1050 O: 0-30 35Cl: 0-8 37Cl: 0-8

Minimum:				-1.5				
Maximum:		5.0	5.0	100.0				
Mass	Calc. Mass	mDa	PPM	DBE	i-FIT	Norm	Conf(%)	Formula
399.0404	399.0394	1.0	2.5	10.5	1084.5	n/a	n/a	C19 H17 O5 37Cl2

Fig. S28. The design of rice seedling-pot arrangement in the greenhouse.

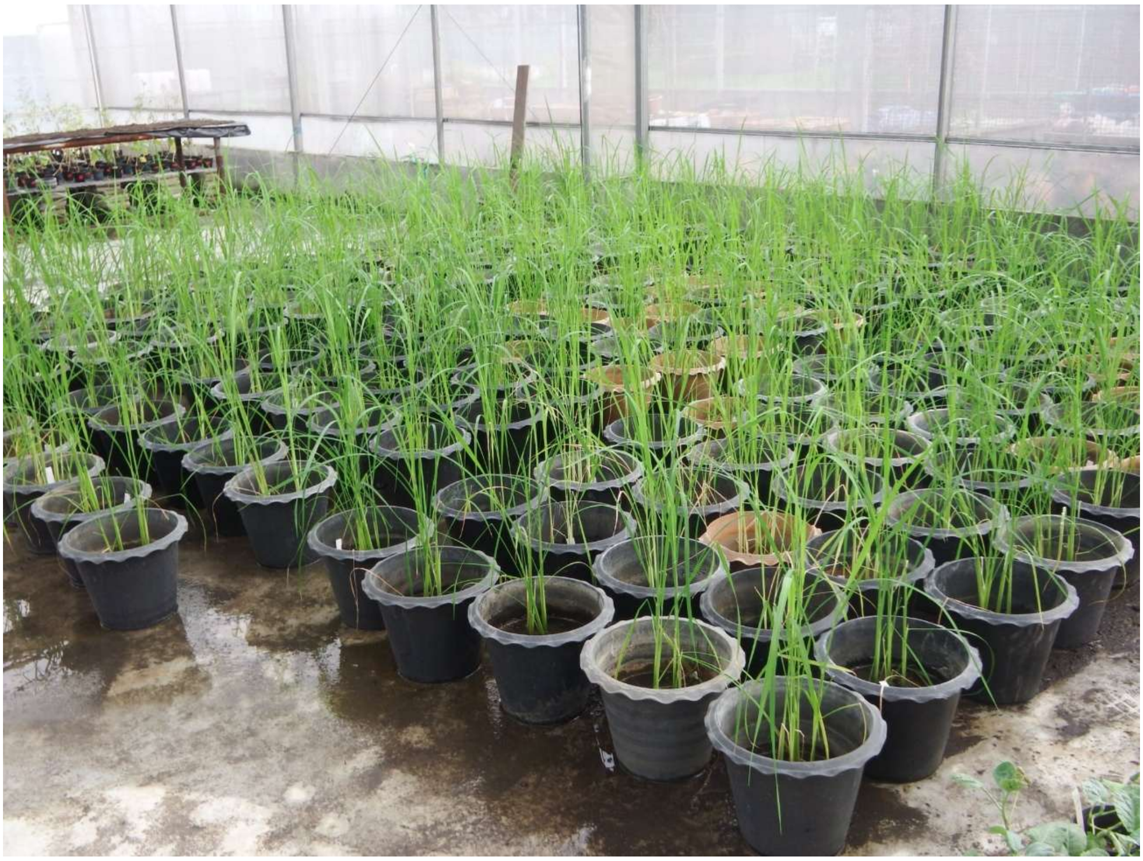

Supplement: Supplementary file 1 [file marinedrugs-23-00461-s001.zip › marinedrugs-3938988-supplementary.pdf]
